# LRIG1 is a gatekeeper to exit from quiescence in adult neural stem cells

**DOI:** 10.1038/s41467-021-22813-w

**Published:** 2021-05-10

**Authors:** María Ángeles Marqués-Torrejón, Charles A. C. Williams, Benjamin Southgate, Neza Alfazema, Melanie P. Clements, Claudia Garcia-Diaz, Carla Blin, Nerea Arranz-Emparan, Jane Fraser, Noor Gammoh, Simona Parrinello, Steven M. Pollard

**Affiliations:** 1 MRC Centre for Regenerative Medicine & Cancer Research UK Edinburgh Centre, University of Edinburgh, Edinburgh, UK; 2 Samantha Dickson Brain Cancer Unit, UCL Cancer Institute, University College London, London, UK; 3 Cancer Research UK Edinburgh Centre, Institute of Genetics and Molecular Medicine, University of Edinburgh, Edinburgh, UK

**Keywords:** Growth factor signalling, Neural stem cells, Quiescence

## Abstract

Adult neural stem cells (NSCs) must tightly regulate quiescence and proliferation. Single-cell analysis has suggested a continuum of cell states as NSCs exit quiescence. Here we capture and characterize in vitro primed quiescent NSCs and identify LRIG1 as an important regulator. We show that BMP-4 signaling induces a dormant non-cycling quiescent state (d-qNSCs), whereas combined BMP-4/FGF-2 signaling induces a distinct primed quiescent state poised for cell cycle re-entry. Primed quiescent NSCs (p-qNSCs) are defined by high levels of LRIG1 and CD9, as well as an interferon response signature, and can efficiently engraft into the adult subventricular zone (SVZ) niche. Genetic disruption of *Lrig1* in vivo within the SVZ NSCs leads an enhanced proliferation. Mechanistically, LRIG1 primes quiescent NSCs for cell cycle re-entry and EGFR responsiveness by enabling EGFR protein levels to increase but limiting signaling activation. LRIG1 is therefore an important functional regulator of NSC exit from quiescence.

## Abstract (web summary)

How neural stem cells can transition between states of proliferation and quiescence is unclear. Here, the authors identify Lrig1 as a specific marker for the primed quiescent state and demonstrate that Lrig1 maintains cells in a quiescent state via modulation of the EGFR pathway.

## Introduction

Homeostasis in many adult tissues requires the balancing of stem cell proliferation and differentiation. Tissue stem cells often reside in a non-cycling quiescent state. Generation of new cells that are needed for tissue turnover and repair therefore requires tight regulation as cells transit from dormant or slow-cycling states, into rapid proliferation. The mechanisms controlling this fundamental process are not well understood, but once determined may open up new rational therapeutic strategies to control stem cell activity to treat disease or tissue injury^1,2^. Understanding regulation of quiescence is also of importance in oncology, as quiescent cancer cells evade standard cytotoxic therapies and drive regrowth of the tumor.

Neural stem cells (NSCs) are situated within two major neurogenic zones of the adult mouse brain: the sub-granular zone of the hippocampus (SGZ)^3^, and the subventricular zone (SVZ) lining the lateral ventricles^4^. SVZ NSCs are responsible for the production of neuroblasts that migrate along the rostral migratory stream (RMS), destined for terminal neuronal differentiation in the olfactory bulb (OB)^4^. Adult NSCs are heterogeneous with regards to cell cycle status and exist in a range of distinct quiescent and proliferative states that are poorly understood. Quiescent stem cells are by definition non-cycling and lack expression of proliferative markers such as Ki67 and MCM2^5^.

Quiescent adult NSCs express GFAP and CD133/prominin^6–8^. These cells generate the rapidly proliferating amplifying progenitors (type C cells), which are GFAP negative, and express high levels of epidermal growth factor receptor (EGFR) and mitotic markers such as MCM2^9^. NSC quiescence is controlled in vivo by a complex repertoire of signals provided by the SVZ niche^10^. These include local cell-cell interactions, secreted factors from the blood or cerebrospinal fluid, as well as longer range signals from neuronal inputs or factors secreted from the choroid plexus^11–13^. These must all be integrated by the NSCs to regulate cell behavior and fate.

A subset of GFAP-expressing NSCs in the SVZ expresses EGFR, and are actively cycling. These have been termed activated NSCs^9^. Indeed, EGF is a key mitogen for NSCs, both in vitro and in vivo^9,14–17^. Despite the complex array of signals within the NSC niche, candidates for regulating NSC fate have emerged, including BMPs, FGFs, and EGF signaling pathways. In the SVZ, BMP-2 and BMP-4 are secreted predominantly by neural stem and progenitor cells; Noggin is produced by adjacent ependymal cells that line the ventricles^18^. The BMP downstream effector, Id1 is a marker of quiescent NSCs in vivo^19^. This local niche therefore regulates the cell cycle state^14,18,20–22^.

Single-cell RNA-seq analysis has also revealed a continuum of cell states during activation of quiescent NSCs and their transition into rapidly proliferative progenitors^23^. This includes a “primed” quiescent state^23^. However, single-cell snapshots of gene expression cannot provide information on cellular dynamics. Also, clonal lineage tracing of NSC cell fate in the adult SVZ in vivo is challenging. It has therefore remained unclear whether primed GFAP^+^EGFR^+^ cells exist transiently, or instead represent a distinct and stable cell type. It also is difficult to dissect key biochemical regulatory processes in vivo, as these cell populations are rare and located deep in the adult brain. How do activated NSCs emerge from their more dormant ancestors? Do they have a unique transcriptional identity and regulatory apparatus? What mechanisms control EGFR activation and cell cycle re-entry?

Exposure of proliferating mouse NSCs to BMP-4 in culture causes cells to rapidly exit the cell cycle, downregulate NSC markers (e.g. Nestin) and upregulate astrocyte markers such as GFAP^24^, leading to the suggestion that BMP triggers terminal astrocyte differentiation. However, in vivo type B quiescent NSCs also express GFAP^7^ and can be low or negative for Nestin^25^. BMP-induced astrocytic cells may therefore be dormant NSCs, rather than terminally differentiated astrocytes as originally thought. Indeed, BMP4 triggers quiescence in adult NSCs both in vitro and in vivo^26–28^, but it remains unclear whether this requires co-stimulation of other pathways such as FGF signaling^28^ and whether distinct quiescent states are induced.

Here, we therefore explored the differences between NSCs cultured in BMP4 alone and BMP4/FGF2. We hypothesized that a distinct primed quiescent NSC state may be imposed by BMP with FGF-2 combined, whereas BMP may induce a deeper quiescent state. We report culture conditions that can capture distinct quiescent states. By cross-comparison of these distinct cell states we uncover defining molecular features of each. LRIG1, a negative regulator of RTK signaling^29^, emerges as a critical gatekeeper of the exit from quiescence. LRIG1 enables increases in EGFR protein, but constrains activation of signaling. Genetic ablation of *Lrig1* in NSCs in vivo leads an increase of proliferation. In this way, the *Lrig1*-expressing quiescent NSCs become primed, and are poised for cell cycle re-entry and subsequent transition into the activated and proliferative NSCs.

## Results

### Quiescent NSCs in vivo can be subdivided into putative deep and shallow quiescent states using a Fucci2a reporter and CD9

The Fucci system was developed to enable live cell visualization of cell cycle, as it contains fluorescent proteins with cell cycle stage-dependent degrons^30^. To understand the diversity of quiescent NSC states in the adult mouse brain, we first characterized the SVZ of Fucci2a reporter mice^31^. Improved Fucci2a mice were developed by Mort et al., and contain a bicistronic cassette targeted to the *Rosa26* safe harbor encoding aVenus-hGem and mCherry-hCdt1 linked by a T2A self-cleaving peptide sequence^31^. These enable monitoring of distinct cell cycle phases: early G1 or G0 (black/low red), late G1 or shallow G0 (high red), G1/S (yellow) and S/G2/M phase (green).

**Fig. 1:**
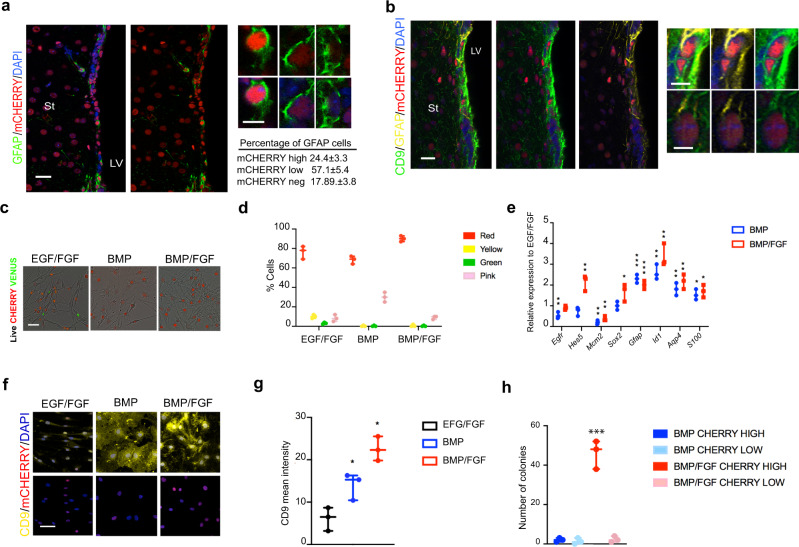
Quiescence NSCs express high levels of the Cdt1 red Fucci reporter in vivo. **a** Immunohistochemistry for GFAP (green) in the SVZ. mCherry Fucci reporter (red) and nuclear counterstaining with DAPI (blue). Right, detail of GFAP positive cells expressing different levels of Fucci red reporter and quantification (*n* = 3 independent mice). **b** Immunohistochemistry for GFAP (yellow), CD9 (green), mCHERRY Fucci reporter (red), and nuclear counterstaining DAPI (blue). Right, detail of GFAP positive cells with different levels of CD9 and mCherry Fucci reporter. Nuclear counterstaining with DAPI (blue). **c** Live imaging of the Fucci NSCs treated with EGF/FGF, BMP and BMP/FGF showing mCHERRY (red) and VENUS (green) reporters. **d** Flow cytometry Fucci quantification of the percentage of cells positive for Cdt1-mCherry (red) and VENUS (green), negative or low levels of fluorescence (pale pink) and double positives for both reporters (yellow) (*n* = 3). **e** qPCR for different markers (*Egfr, Hes5, Mmc2, Sox2, Gfap, Id1*) in the different conditions (data relative to EGF/FGF) (*n* = 3). **f** Immunocytochemistry for CD9 (yellow) in Fucci NSC line, mCherry (red) and nuclear counterstaining with DAPI (blue) (*n* = 3 independent NSC lines). **g** Quantification of the CD9 mean intensity in NSCs treated for EGF/FGF2, BMP4, and BMP/FGF2 for 3 days. Quantification by Fiji (*n* = 3 independent experiments per condition, average intensity of min 500 cells per group). **h** Number of colonies in the sorted population of Fucci cell cycle reporter based high levels of CD9 and mCHERRY levels (h-high, l-low) (*n* = 3). Data are shown as mean ± SEM of the indicated number of the experiments (*n*) (*p < 0.05; **p < 0.01; ***p < 0.001). St:striatum, LV: Lateral Ventricle. Scale bar in (**a**, **b**) is 20 μm, (**c**, **f**) is 50 μm. Scale in details:10 μm. Source data are provided as a Source Data File.

Surprisingly, during the characterization of the adult SVZ from Fucci2a reporter mice we uncovered an unexpected heterogeneity in the levels of the mCherry-Cdt1 reporter in the GFAP populations (Fig. 1a) (mCherry-Cdt1^high^ levels: 24.5%; low levels: 57.1%; and negative: 17.3%; *n* = 3). The subset of GFAP-expressing cells that were brightest for mCHERRY, also co-expressed CD9 (Fig. 1b), which distinguishes NSCs from parenchymal astrocytes and is a marker of a subset of activated qNSCs (type B cells)^23^. CD9 expression was detectable in 82.04 ± 2.3% of the total GFAP population. We scored high and low levels of CD9 in the GFAP population and only 20% of the GFAP population express high levels of CD9. These highest CD9 expressing cells also co-expressed the highest mCherry levels. We analyzed the Fucci2a SVZ by immunocytochemistry for the proliferative marker KI67 and the mCHERRY reporter but could not identify any GFAP^+^KI67^+^ cells with high levels of mCherry; from the GFAP^+^Ki67^+^ population only 10.66 ± 4.04% displayed low levels of mCherry and 89.33 ± 4.04 were negative for the red reporter (Supplementary Fig. 1a). Cells with highest levels of mCherry were also negative for S100B, consistent with a qNSC identity (Supplementary Fig. 1b)^32^. We found ependymal cells lining the ventricle and S100B^+^ expressed high mCherry. Our results suggest that qNSCs in vivo can be subdivided based on the levels of mCherry-Cdt1 Fucci reporter and levels of the surface marker CD9 and these might mark distinct G0 or quiescent states.

As our goal was to dissect the regulatory mechanisms that control qNSC exit from quiescence we focussed on in vitro analysis, where the biochemical signaling pathways, transcriptional and clonal proliferative capacity can be experimentally dissected. We derived proliferative NSC cultures from the Fucci reporter mice (expanded in EGF/FGF-2). Fucci NSCs cultures displayed all the key markers (Fig. 1e), morphology and differentiation capacity as previously reported for adherent NSCs (Fig. 1c)^24^.

To induce quiescence, we withdrew EGF and exposed cells to either BMP or combined BMP/FGF. In both conditions cells exit cycle and acquire astrocytic morphology (Fig. 1c). After 3 days we determined reporter levels using flow cytometry (Fig. 1d). As expected, under proliferating conditions (EGF/FGF), a full range of cell cycle stages was indicated by the Fucci reporter (Fig. 1c, d). In both BMP and BMP/FGF we did not detect any Venus-hGem expressing (S/G2/M) as cells are not cycling (Fig. 1d). However, there was a significant heterogeneity in the proportions and levels of the mCherry-Cdt1 with significantly higher levels in BMP/FGF compared to BMP alone. Similar to the in vivo situation, the mCherry-hCdt1^high^ cells in BMP/FGF co-expressed high levels of CD9 (Fig. 1f, g) and upon sorting and replating into EGF/FGF conditions this double positive subpopulation contained all of the NSC colony-forming activity (Fig. 1h and Supplementary Fig. 1c). BMP/FGF culture conditions therefore seem to induce a primed qNSC state (p-qNSCs) that is in a shallow quiescence, whereas in BMP alone the cells correspond to a more dormant quiescent qNSC state (d-qNSCs).

### Proliferative NSCs can be directed into distinct quiescent states in vitro

**Fig. 2:**
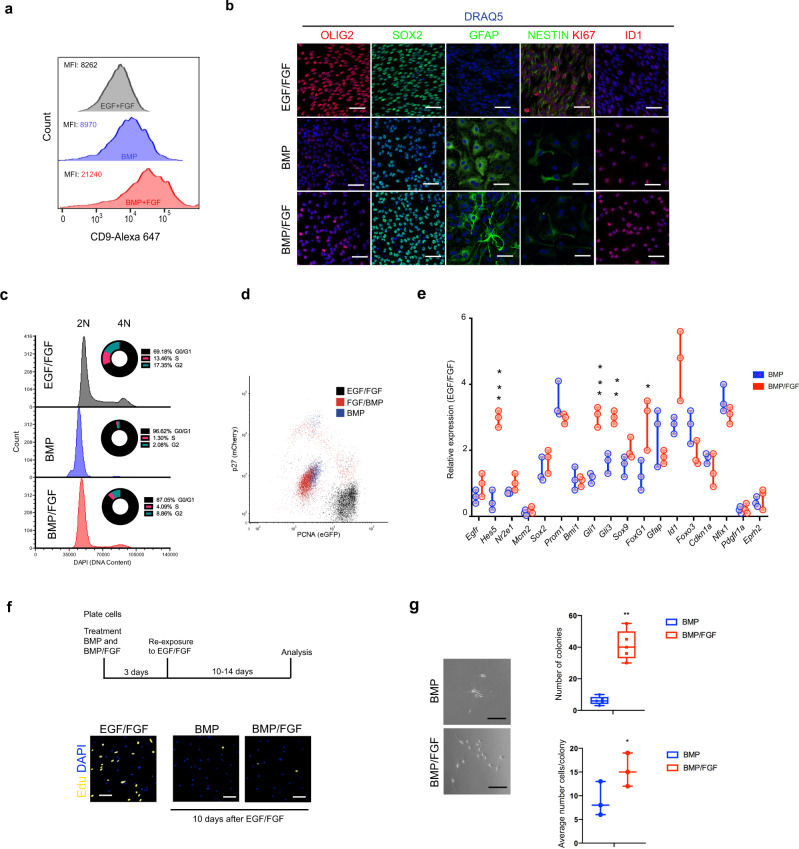
BMP and FGF2 condition drives a quiescence reversible state in NSCs. **a** Histogram of cytometry quantification of CD9 in the different conditions MFI (median fluorescence intensity) (*n* = 4). **b** Immunostaining of NSCs treated with EGF/FGF, BMP, and BMP/FGF for 3 days. OLIG2 (red), SOX2 (green), GFAP (green) NESTIN (green) KI67 (red) and ID1 (red). Nuclear counterstaining in blue with DRAQ5. Images showing ICC in group of cells to appreciate staining (*n* = 5). **c** Quantification of the DNA content using DAPI and flow cytometry (*n* = 3). **d** Cytometry analysis of double knock-in mCherry-p27 and eGFP-PCNA NSC line in the different conditions (*n* = 4). **e** Quantification of the relative expression of different genes in the cells treated with BMP and BMP/FGF2 (*n* = 3). **f** EdU incorporation images (yellow) in the NSCs in presence of EGF/FGF. Nuclear counterstaining with DAPI (blue). Quantification of percentage of EdU positive cells, during the treatment (left), and after to re-exposure to mitogens (right) (*n* = 3). **g** (top) Phase contrast images of the colony-forming assay of the cells treated with BMP and BMP/FGF (bottom). Quantification of the number of colonies after to re-exposure to mitogens (*n* = 5). Scale bar in (**b**, **f**, **g**) is 50 μm. Mean is indicated in the box and whiskers plots from minimum to maximum. Data are shown as mean ± SEM of the indicated number of the experiments (*n*) (∗*p* < 0.05; ∗∗*p* < 0.01; ∗∗∗*p* < 0.001). Source data are provided as a Data Source File.

To extend the findings from the Fucci NSCs we focused on an independent wild-type NSC line derived from C57BL6 adult brain, which has no fluorescent reporter transgenes. The BL6-NSCs were karyotypically normal^33^ and expressed standard NSC markers (Fig. 2b). Using flow cytometry we found, similarly to the Fucci-NSC results, the levels of CD9 protein were highest in BMP/FGF treatment relative to either BMP alone or EGF/FGF (proliferating NSCs) (Fig. 2a). Moreover, there were similar differential cellular phenotypes; the BMP/FGF was clearly more elongated, with more compact and rounded nuclei. SOX2 is a key NSC marker, and in BMP/FGF we observed increased protein levels in each cell (similar to EGF/FGF) and more homogeneous across the cell population (Fig. 2b). We analyzed the cell cycle of the different conditions using DNA content (Fig. 2c) and we did not find any significant difference in cell cycle between BMP alone and BMP/FGF.

In both BMP and BMP/FGF conditions qNSCs retained expression of markers associated with Notch signaling and Shh signaling (Hes5, Gli1, Gli3) as well as Sox9, FoxG1, and Id1^19,25,34–36^ (Fig. 2e). However, in the BMP/FGF condition these were typically higher (Fig. 2e). p21 (*Cdkn1a*) was not increased, suggesting these are not driven into senescence (Fig. 2e). To assess functional differences between the BMP and BMP/FGF-induced states, we tested their ability to re-enter the cell cycle upon return to EGF/FGF. Both EdU incorporation (Fig. 2f) and NSC colony formation assays indicated that BMP/FGF treated cells were more readily able re-enter cell cycle (Fig. 2f, g).

Fucci reporters cannot distinguish G0 and G1. We therefore next explored the candidate regulator p27 (encoded by *Cdkn2b*), which is thought to mark G0^37,38^. BMP cells were found to be very high for p27 protein using immunocytochemistry compared to BMP/FGF, and therefore in a deep G0 state (Supplementary Fig. 1f, g). In vitro generated quiescent NSCs are therefore being driven into a p27-expressing G0 state. However, the higher p27 levels in BMP alone versus BMP/FGF suggested deeper quiescence, consistent with the differential responses to EGF/FGF in our colony formation experiments (Fig. 2f). To extend the characterization of cell cycle markers, we created a double knock-in mCherry-p27 and eGFP-PCNA reporter NSC line. We found that NSCs in EGF/FGF, displayed high levels of PCNA and are negative for p27 (Fig. 2d and Supplementary Fig. 1d–g). BMP and BMP/FGF treated cells become cell cycle arrested and consistently have activated p27 but low PCNA (Fig. 2c and Supplementary Fig. 1d–g). However, the putative primed NSCs in BMP/FGF have lower p27 levels than in BMP alone, consistent with them being in a shallower or primed state (Fig. 2d).

Quiescent NSCs in vivo have previously been shown to have reduced rates of translation^17,18^. Using O-propargyl-puromycin (OP-Puro) incorporation assays we confirmed that BMP or BMP/FGF treated cells did indeed have reduced levels of translation (Supplementary Fig. 2b, c). Altogether, the above data suggest we can model two distinct types of quiescence: p-qNSCs and d-qNSCs.

### Primed quiescent NSCs transplanted in vivo can engraft into the SVZ and re-engage in neurogenesis

If the in vitro generated p-qNSCs are a physiologically relevant culture model, then following orthotopic transplantation we might anticipate engraftment in vivo into the niche and reconstitution of neurogenesis. Previously, transplantation of acutely isolated, or primary cultured neurospheres, have focused on their differentiation capacity (e.g., by transplanting into the cortex or striatum). However, Neumeister et al. did demonstrate that short-term cultured neurospheres can indeed engraft into the niche and self-renew^39^. This may be related to the tropism of transplanted NSCs to the niche in slice cultures or when injected into the SVZ^39,40^, something we have also observed in whole brain slice cultures^41^. We therefore reasoned that upon transplantation in vivo in live animals in regions adjacent to the SVZ, d-qNSCs, and p-qNSCs may home to the niche and re-engage in their differentiation programs.

**Fig. 3:**
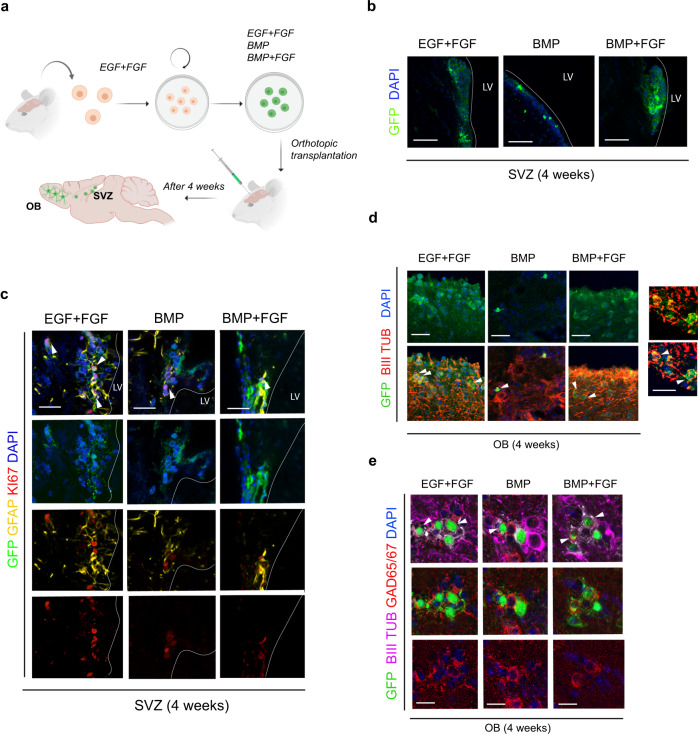
Quiescence NSCs allow long term regeneration. **a** Schematics of the experiments. NSCs derived from mouse, after several months of expansion in vitro are transplanted in the SVZ for 1 month. **b** Panoramic pictures of the NSCs (GFP, green) in the SVZ after 1 month. NSCs were treated with EGF/FGF, BMP-4, and FGF/BMP-4 previously to be transplanted. **c** Immunostaining for GFP (green), GFAP (yellow), Ki67 (red) nuclear counterstaining with DAPI (blue). **d** Immunostaining for GFP (green), BIII TUBULIN (red), DAPI (blue) showing neurons arriving to the OB. **e** Immunostaining for GFP (green), BIII tubulin (magenta) and GAD65/67 (red) and nuclear counterstaining with DAPI (blue). LV: Lateral Ventricle. OB: Olfactory Bulb. Detail of co-staining GFP and BIII TUBULIN (red). Scale bar in (**b**) is 100 μm, (**c**, **d**) is 50 μm and in (**e**) is 20 μm. Scale bar in detail is 20 μm (*n* = 3 analyzed transplanted mice per condition). Schematics in (**a**) were generated using BioRender software.

We transplanted orthotopically ~2000 cells that had been cultured for 3 days in EGF/FGF, BMP/FGF, or BMP alone into the adult SVZ region of syngeneic C57/BL6 mice (Fig. 3a). Transplanted NSCs were stably transfected with an eGFP-expression cassette enabling tracking of their progeny. After one month, the SVZ and olfactory bulbs were dissected characterized using an anti-GFP antibody. We quantified the number of GFP cells per section in each condition of transplant and in the SVZ we found 87%±12, 35%±8, 55%±13 in EGF/FGF, BMP, and BMP/FGF respectively. In the OB the number of GFP cells per section was 36%±7, 14%±3.6, 21%±3.2 (*n* = 3 animals per condition, 3 brain slices per animal in the injection point). GFP-positive cells were clearly identifiable and engrafted successfully within the SVZ (Fig. 3b) and many of these remained proliferative (%GFAP/Ki67 37 ± 4.3, 24 ± 5.5 and 26.3 ± 6, treated previously in EG/FGF, BMP, and FGF/BMP, respectively) (Fig. 3c). GFP^+^ cells were detected along the RMS (Supplementary Fig. 3a). GFP^+^ neuronal-like cells were also found within the OB that expressed beta-III Tubulin (Fig. 3d) and interneuron marker GAD65/67 (Fig. 3e). p-qNSCs can therefore recommence neurogenesis following transplantation.

The efficiency of engraftment was clearly reduced for the d-qNSCs compared to p-qNSCs and only a small number of neuronal cells were identified in the OB (Fig. 3d). p-qNSCs therefore appear more primed for re-engraftment and neuronal production. Proliferative NSCs grown in (EGF/FGF) were also able to engraft into the niche and re-enter neurogenesis, confirming the interconvertibility of quiescence and proliferative states (Fig. 3b, d). These in vivo potency assays support our hypothesis that BMP/FGF cultured NSCs are physiological relevant models and can therefore be used to dissect pathways regulating entry and exit from quiescence.

### Increased levels of *Lrig1* and an interferon response signature distinguish dormant and primed quiescent NSCs

**Fig. 4:**
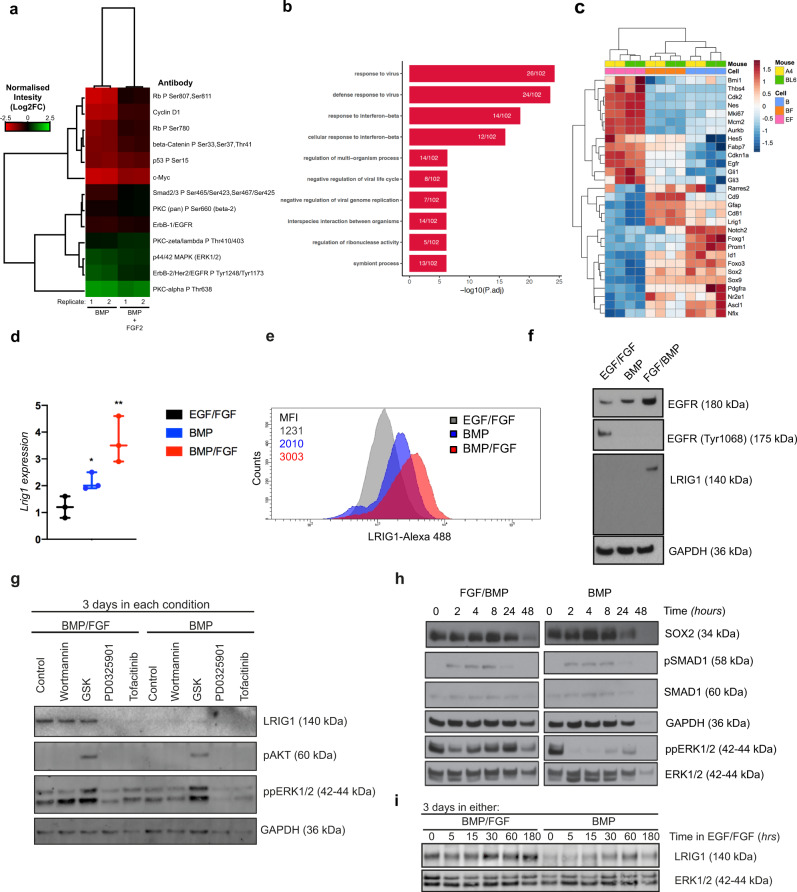
Dormant and primed quiescent NSCs have distinct signaling pathways and transcriptional programs. **a** RPPA data analysis of the NSCs in BMP and BMP/FGF (*n* = 3). **b** The top biological GO terms in biological process enriched genes in BMP/FGF condition. **c** Heatmap showing the top significant genes. **d** *Lrig1* expression by QPCR in the different conditions (*n* = 3). **e** Histogram of LRIG1 expression by cytometry (*n* = 3). Quantifications show mean fluorescent intensity (MFI). **f** Immunoblot of LRIG1, EGFR (total), and EGFR-P. Loading control GAPDH. **g** Immunoblot for LRIG1, pAKT, ppERK1/2, GAPDH in NSCs treated with BMP and BMP + FGF for 3 days (*n* = 3). Different kinases inhibitors were used (Wortmannin, GSK, PD0325901, Tofacitinib) (*n* = 3). **h** Immunoblot for SOX2, pSMAD1, SMAD1, GAPDH, ppERK1/2, ERK in NSCs treated with BMP and BMP/FGF for 3 days and re-exposure to EGF + FGF, different time exposures (*n* = 3). **i** Immunoblot for Lrig1 in NSCs treated with BMP/FGF and BMP and re-exposure to EGF/FGF in a time course. Loading control, ERK1/2 (*n* = 3). Data are shown as mean ± SEM of the indicated number of the experiments (*n*) (∗*p* < 0.05; ∗∗*p* < 0.01). Source data are provided as a Source Data File.

The striking functional differences seen between d-qNSC and p-qNSC in transplantation encouraged us to perform a more extensive characterization of transcriptional and signaling pathways that differ between these two cell states. Reverse phase protein array (RPPA) were used to assess 62 proteins and phosphoproteins of major signaling pathways and suggested that p-qNSCs express higher levels of cell cycle markers relative to BMP alone, such as CYCLIN D1 and its phosphorylated target RB-P (Ser780), and increased levels of MYC (Fig. 4a). They also display slightly higher levels of cMYC and EGFR (ErbB-1). This is consistent with the Fucci2a reporter experiments described above and further indicates these are in a state primed for cell cycle re-entry and EGFR responsiveness.

We next performed RNA-seq to identify transcriptional differences associated with d-qNSCs versus p-qNSCs. Initial PCA analysis and clustering confirmed that each cell state could be clearly distinguished (Supplementary Fig. 4a). Unexpectedly, however, the only gene ontology terms enriched in the p-qNSCs were those related to immune regulation; specifically, interferon signaling (Fig. 4b). Indeed, the top 40 most significantly differentially expressed genes, included established immune regulators (*Oasl2, Mx2, Bst2, Lgals9,* and *Rsad2*) (Supplementary Fig. 4b). We noted that many of these genes are part of a previously reporter interferon-related damage response signature (IRDS)^42^, including *Bst2, Ifi44, Ifit1, Ifit3, Irf7, Mx2, Oals2, Usp18* (Supplementary Fig. 4b). We note that interferon response signatures were identified in single-cell analysis of injured SVZ^23^, but the functional significance of this remains unclear.

In addition to this signature, there were many other notable genes that were differentially expressed between d- and q-NSCs. Most notably, the transmembrane protein LRIG1, which interacts with ErbB family and reduces signaling strength by negatively regulating both protein levels and activity^43^, showed higher levels in p-qNSCs compared to d-qNSCs. LRIG1 is also known to be a quiescence regulator in other tissues such as the intestine and skin^42,44^. A recent publication has described the expression of Lrig1 in the SVZ^29^, but has not been functionally explored in the regulation of qNSCs, despite EGFR signaling being critical to their self-renewal. We therefore focused our attention in exploring whether LRIG1 is a critical functional regulator that explains the distinct dormant and primed quiescent NSCs and is involved in exit from quiescence into proliferation.

We confirmed that *Lrig1* mRNA levels are increased within p-qNSCs compared to d-qNSCs (Fig. 4d). Flow cytometry confirmed that LRIG1 protein was also increased (Fig. 4e) and western blotting confirmed higher levels of the protein within the BMP/FGF condition (Fig. 4f). Reduced levels of EGFR Tyr1068 phosphorylation were noted in this condition, indicating reduced EGFR activation/signaling (Fig. 4f). Also, d-qNSCs (treated with BMP4) can upregulate LRIG1 when exposed to FGF, consistent with them shifting into the p-qNSC state (adding BMP4/FGF2) (Supplementary Fig. 4c). LRIG1 expressing cells also co-expressed high levels of Cdt1-mCherry, CD9 and SOX2 (Supplementary Fig. 4d) indicating that *Lrig1* expression correlates with the colony-forming quiescent subpopulation we had defined earlier. We conclude that LRIG1 is a candidate functional regulator of the transition from dormancy into a state primed for EGFR responsiveness and cell cycle re-entry.

### FGF-stimulated MAPK signaling controls *Lrig1* expression

The increased levels of Lrig1 detected in BMP/FGF versus BMP alone, prompted us to explore signaling responsiveness in each condition, and determine potential downstream signaling pathways that might explain their different potency. To determine which signaling pathways sustain LRIG1 levels we used different pharmacological inhibitors of kinases associated with candidate signaling pathways (Wortmanin, PI3K; GSK690693, AKT; PD0325901, MEK1/2; Tofacitinib, JAK/STAT. Inhibitors of MEK1/2 (PD0325901), and JAK1/2 (Tofacitinib), were each able to reduce LRIG1 protein levels and these are upstream of the ERK1/2 signaling and JAK/STAT signaling respectively (Fig. 4g). Interplay between these pathways may be therefore mediate FGF-induced increases in LRIG1; however, we note that pAKT levels are increased in BMP/FGF (Supplementary Fig. 4e).

We also determined the signaling flux after return to EGF/FGF-2 by monitoring the downstream effectors, phospho-ERK1/2 (Thr Thr202/Tyr204) (mitogen-activated protein kinase superfamily) and phospho-SMAD1 (Ser463/465) (an effector of BMP signaling). We found that the duration of ERK1/2 phosphorylation (i.e. MAPK signaling output) is significantly extended in BMP/FGF. By contrast, activation of p-SMAD1 had similar levels and kinetics in each condition indicating levels of BMP signaling are not altered (Fig. 4h). These data indicate that it is the ability to rapidly initiate and sustain MAPK signaling that likely facilitates cell cycle re-entry in p-qNSCs compared to d-qNSCs.

### LRIG1 regulates the entry and exit from quiescence in NSCs in vitro

**Fig. 5:**
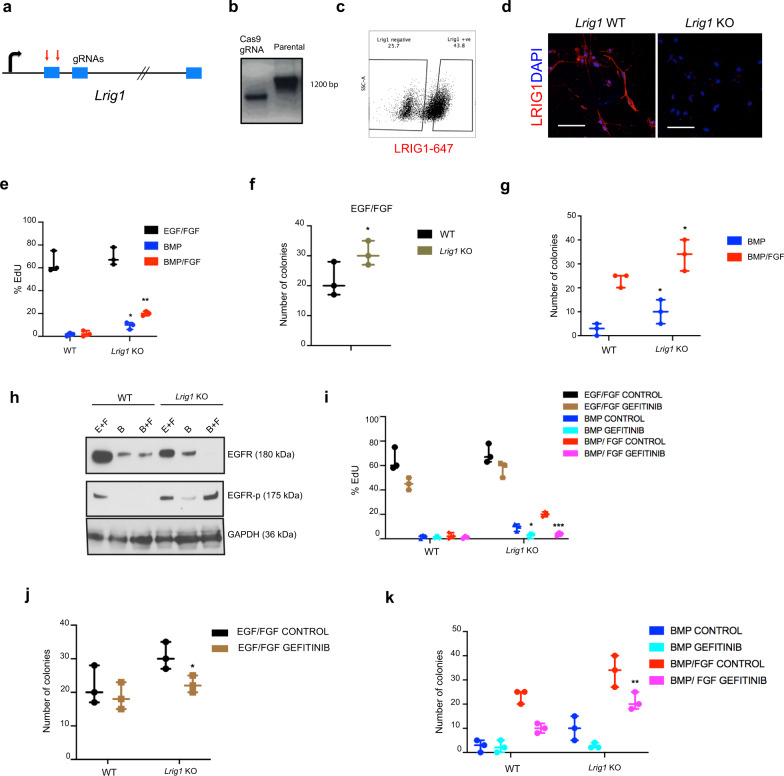
LRIG1 is necessary to enter in quiescence state. **a** Schematics of *Lrig1* gene disruption. **b** PCR confirming CRISPR-Cas9 (control and parental line). **c** Flow cytometry plots with the transfected NSCs. **d** ICC for LRIG1(red) and nuclear counterstaining with DAPI (blue) in sorted populations after transfection. **e** EdU quantification of the WT and *Lrig1* KO NSCs in the different conditions (EGF/FGF2, BMP, and BMP/FGF2) (*n* = 3). **f** Quantification of the single-cell colony formation in the WT and *Lrig1* KO cells in EGF/FGF2 (*n* = 3). **g** Quantification of the number of colonies of deficient and WT NSCs after the treatment with BMP and BMP/FGF2, after to re-exposure to mitogens (*n* = 3). **h** *Lrig1* KO cells maintain high levels of pEGFR; WB of EGFR total and EGFR-p in WT and *Lrig1* KO cells in the different conditions (EGF/FGF2, BMP and BMP/FGF2). Loading control GAPDH. **i** Quantification of the EdU positive cells in KO and control cells in the different conditions using Gefitinib (*n* = 3 per condition). **j** Single-cell colony-forming assay of the deficient and control NSCs using Gefitinib (*n* = 3 per genotype and condition, 48 single cells plated in each group each time). **k** Quantification of the colony formation of the WT and *Lrig1* KO NSCs in each condition (BMP and BMP/FGF2) using EGFR inhibitor (Gefitinib) (*n* = 3 per condition). Scale bar in *d* = 50um. Data are shown as mean ± SEM of the indicated number of the experiments (*n*) (∗*p* < 0.05; ∗∗*p* < 0.01; ∗∗∗*p* < 0.001). Source data are provided as Source Data File.

To explore if *Lrig1* has a functional role in p-qNSCs we generated *Lrig1* mutant NSCs using CRISPR/Cas9^45^ (Fig. 5a). PCR genotyping confirmed CRISPR/Cas9 gRNAs were active in our NSCs and successful mutation of *Lrig1* (Fig. 5b). An LRIG1 antibody was used to isolate LRIG1-negative cells by FACS (Fig. 5c). Loss of LRIG1 protein was confirmed by immunocytochemistry in these sorted populations (Fig. 5d). We tested the differentiation capacity in these cells and found similar multipotency potential to the parental control NSCs (Supplementary Fig. 5b).

Using *Lrig1* mutant NSCs we next asked whether there was an impact on the ability to enter or exit quiescence. In either BMP-4 or BMP/FGF, *Lrig1* mutant cells proliferate more than parental controls (Fig. 5e and Supplementary Fig. 5c), suggesting they are not able exit cell cycle. After re-exposure EGF/FGF-2, the cells were able to form more significantly more colonies than their controls (Fig. 5g). Restoration of LRIG1 in mutant NSCs (Mouse Lrig1 FLAG-tagged, expressed from CMV promoter) rescued the ability to enter quiescence in both BMP and BMP/FGF2 treatments (Supplementary Fig. 5a). Further analysis of the EGFR levels and signaling activity revealed that *Lrig1* mutant cells treated with BMP or BMP/FGF retain active EGFR signaling (EGFR Y1068), suggesting that loss of LRIG1 limits the ability to suppress autocrine EGFR activation in NSCs (Fig. 5h). Following Gefitinib treatment (an EGFR inhibitor), the Lrig1 mutant cells ceased proliferation (Fig. 5i–k; and Supplementary Fig. 5c, d). LRIG1 therefore seems to control the entry and exit of quiescence through EGFR.

To demonstrate in vivo function, we next tested the potency of *Lrig1* mutant cells following transplantation into the adult SVZ. One month after transplantation *Lrig1* mutant cells were identified successfully engrafted into the SVZ to a similar extent as parental controls in each condition (Supplementary Fig. 6a) (BMP, BMP/FGF or EGF/FGF). We quantified the % of GFP cells per section in each condition of transplant and in the SVZ we found in the *Lrig1* KO transplants 83 ± 15, 60 ± 9, 76 ± 5 (EGF/FGF, BMP4, BMP/FGF respectively) vs 79 ± 10, 37 ± 9, 53 ± 11 (EGF/FGF, BMP4, BMP/FGF respectively) in the *Lrig1* WT ones. However, in each condition there was an increased proliferation in the *Lrig1* mutant cells (Supplementary Fig. 6b). Cells lacking *Lrig1* therefore cannot respond appropriately to signals within the SVZ niche. We conclude that LRIG1 is an important functional regulator that constrains proliferation and EGF signaling in p-qNSCs.

### *Lrig1* overexpression in NSCs induces cell cycle exit

**Fig. 6:**
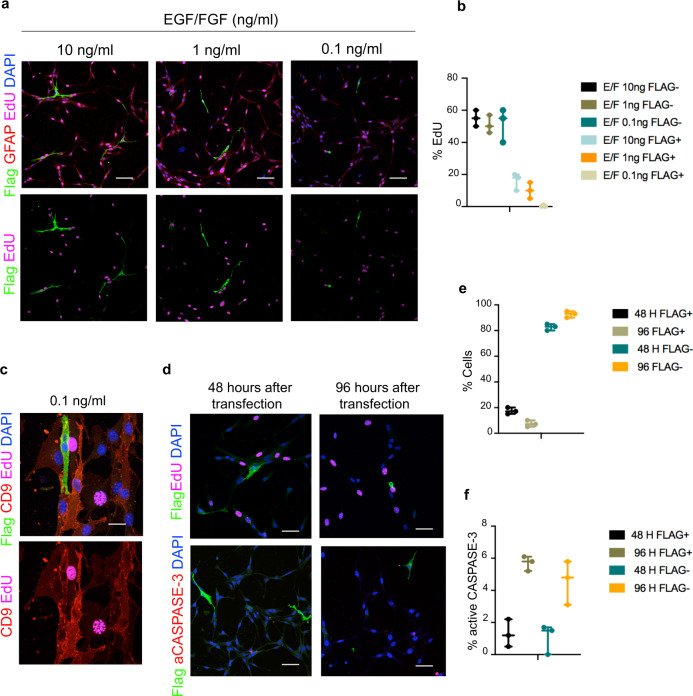
*Lrig1* overexpression in NSCs induces cell cycle exit. **a** IHC for FLAG (green), and GFAP (red). Detection of EdU (magenta) and nuclear counterstaining in blue (DAPI) with NSC overexpressing *Lrig1* in different concentrations of mitogens (10, 1, and 0.1 ng/ml of both EGF/FGF). **b** Quantification of EdU positive cells after 2 h of pulse in the FLAG + and FLAG- populations. **c** IHC for CD9 (red) and FLAG (green). Detection of EdU in magenta and nuclear counterstaining with DAPI (blue). **d** IHC of FLAG (green), active CASPASE-3 (red). Detection of EdU (magenta) and nuclear counterstaining with DAPI (blue). Cells analyzed 48 and 96 h after transfection. **e** Quantification of EdU positive cells in the FLAG + and FLAG- population after 48 h and 96 h of transfection. **f** Quantification of active CASPASE-3 after 48 h and 96 h of transfection in FLAG + and FLAG- populations (*n* = 3 independent transfections, minimal number of 500 cells per condition). Scale bar in (**a**) is 50 μm, (**c**) is 10 μm and (**d**) is 30 μm. Data are shown as mean ± SEM of the indicated number of the experiments (*n*). Source data are provided as Source Data File.

To investigate the role of increased levels of LRIG1 in the regulation of adult NSCs, WT NSCs were transfected with m*Lrig1*-FLAG, and exposed to each growth factor condition to analyze proliferative responses. NSCs were plated in reduced concentrations of mitogens (as these are normally saturating in standard culture media; we used: 10, 1, 0.1 ng/ml of EGF and FGF2) and after 48 h of transfection and a pulse of 2 h of EdU was given to the cells. FLAG-positive and FLAG-negative cells both displayed low levels of GFAP (Fig. 6a). Some FLAG^+^ cells were able to incorporate EdU (16% and 10%) in higher doses of mitogens (10 and 1 ng/ml EGF and FGF); however, there was significant reduction to 0.3% EdU positive cells in 0.1 ng/ml EGF and FGF conditions. By contrast, Flag negative cells maintained similar levels of EdU incorporation at all mitogen concentrations (Fig. 6a, b). Moreover, CD9 was expressed in FLAG + cells that didn’t incorporate EdU (Fig. 6c), suggesting that CD9 is expressed in cells that are not cycling. The increase in LRIG1 levels was not associated with increased apoptosis (Fig. 6d, f). After 96 h following transfection the % of FLAG + cells with respect to the total population (DAPI) was reduced from that at 48 h, consistent with the *Lrig1* overexpressing cells have slower proliferation and being outcompeted (Fig. 6d, e). These data indicate increased LRIG1 can suppress EGF-driven proliferation of NSCs.

### Disruption of *Lrig1* results in activation on NSCs in vivo

**Fig. 7:**
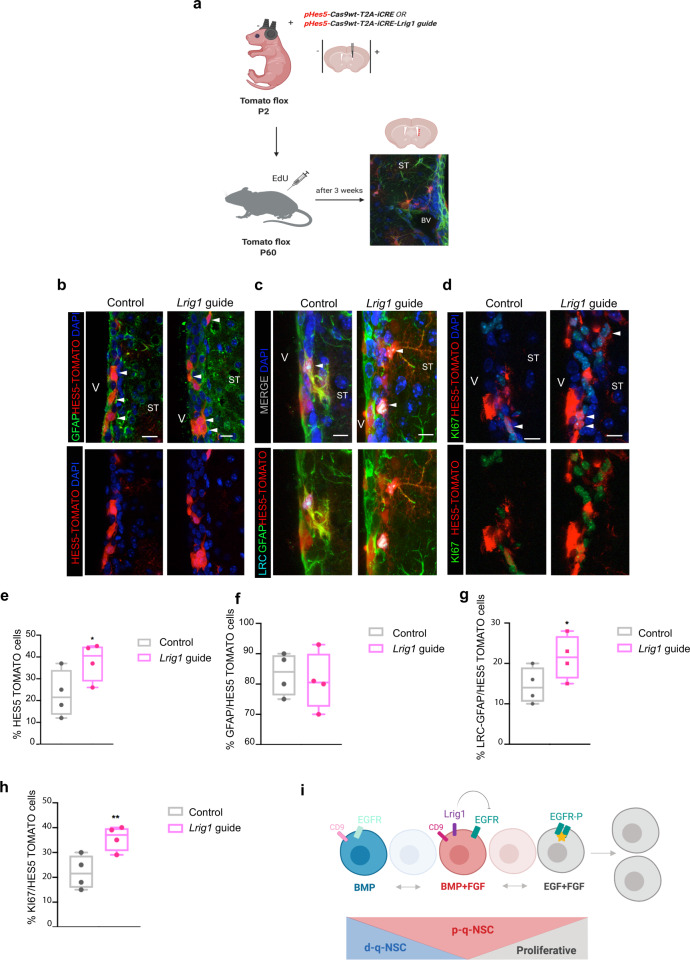
LRIG1 control proliferation in the SVZ. **a** Schematics of the in vivo electroporation procedure. **b** IHC for GFAP (green), Hes5 tomato (red) and DAPI counterstain in mice electroporated with Control plasmid and Lrig1 guide RNA. **c** IHC for LRC (light blue), GFAP (green), and HES5 tomato (red). Nuclear counterstaining with DAPI (blue). **d** IHC for KI67 (green), HES5 TOMATO (red) and nuclear counterstaining with DAPI (blue). **e** Quantification of the number of HES5 tomato positive cells relative to the total ones (DAPI). **f** Quantification of the number of GFAP positive cells in the Hes5 TOMATO population. **g** Quantification of the LRC + in GFAP/HES5 TOMATO population (**h**) Quantification of the percentage of KI67 positive cells in HES5 TOMATO population. **i** Graphical schematics of the results. Scale bar in (**b**, **c**, and **d**) is 10 μm. LV: Lateral Ventricle, ST: Striatum. BV: Blood Vessel (*N* = 4 mice per condition). Mean is indicated in the box and whiskers plots from minimum to maximum. Data are shown as mean ± SEM of the indicated number of the experiments (*n*) (∗*p* < 0.05; ∗∗*p* < 0.01). Schematics in (**a**) are done using BioRender software (original photo of the SVZ). Source data are provided as Source Data File.

To functionally evaluate LRIG1 in the adult SVZ, we genetically ablated *Lrig1* in vivo. Expression of Notch downstream effector *Hes5* has been found to be expressed in quiescence NSCs^16,35^. In addition, we found that *Hes5* was highly expressed in quiescence conditions in vitro (Fig. 2e). We therefore targeted the *Lrig1* gRNAs for conditional expression in *Hes5* expressing cells. We performed in vivo electroporation in postnatal mice and analyzed them after 2 months. We used tomato floxed P2 reporter mice and electroporated a plasmid into the ventricle (p*Hes5*-Cas9wt-T2A-iCRE-*Lrig1*guide and control without guide) (Fig. 7a). To track LRC (labeled retained cells) we injected the mice with EdU and after 3 weeks we sacrificed the mice. After 2 weeks of transfection, we found HES5 tomato positive cells were mostly GFAP positive and some ependymal cells and no DCX is co-located (Supplementary Fig. 7a). In adult SVZ, we analyzed the number of HES5 tomato positive relative to the total number of cells. There were increased numbers of HES5 tomato in the transfected mice with p*Hes5*-Cas9wt-T2A-iCRE-*Lrig1*guide compared to controls (Fig. 7b, c). We found that disruption of *Lrig1* and their control in the Hes5^+^ population exhibited similar proportions of GFAP (Fig. 7b, f). We therefore determined whether this increase in the number of HES5 tomato+ cells was the result of B cell activation. We analyzed LRC + cells in the HES5 + population and we found that NSCs within the *Lrig1* disrupted SVZ displayed more LRC + HES5 + cells than their controls (Fig. 7c, g). Moreover, the SVZ displayed regions of hyperplasia, and the proportion of HES5 + GFAP^+^KI67^+^ cells was increased (Fig. 7d, h).

## Discussion

In this study, we have used the tractable NSC culture models to explore the signaling events, molecular markers and regulatory mechanisms associated with transition of quiescent NSCs into active and proliferative states. Previous single-cell profiling of mRNAs and lineage tracing have suggested a series of transitory states, but it has been difficult to determine those key functional pathways that regulate this process. The in vitro models we developed here have helped to explore these distinct quiescent states and identify LRIG1 as an important regulator of the primed quiescent NSC state. Using our modified in vitro culture conditions (low density BMP versus BMP/FGF) we identified two distinct quiescent NSC states: BMP signaling in the absence of exogenous FGF induces a deep, or dormant, quiescent state; however, when BMP and FGF are combined, a distinct primed quiescent state emerges that can readily re-enter cell cycle.

Previously it has been assumed the increased GFAP expression and loss of NESTIN expression that accompanies BMP treatment of NSCs marks their terminal differentiation to astrocytes^24^. However, this view is inconsistent with our past observations that the BMP treated cells with some low efficiency can revert back into a rapidly proliferating state (Pollard lab, unpublished observations). Our findings in this study, that many stemness markers are retained, suggests BMP signals do not drive terminal astrocyte differentiation, but rather impose a dormant quiescent NSC state, akin to the primed quiescent SVZ (type B cells) that have been reported in vivo.

BMPs have been implicated as regulators of quiescence state in a variety of different adult stem cells including: brain, hair follicle, intestinal, hematopoietic stem cells^18,40,46^. Quiescence is induced by autocrine production of bone morphogenetic proteins (BMPs) in the hippocampus^46^. BMP antagonist Noggin can also induce dormant cells to re-enter the cell cycle, upon which they reacquire neurogenic potential^44^. Also, Fibroblast growth factor 2 (FGF2) is required to suppress terminal astrocytic differentiation and maintain stem cell potency during quiescence state and cooperates with BMP signaling^47^.

By exposing proliferative NSCs to BMP in combination with FGF we identified a G0 state that is primed for cell cycle re-entry. Initially, we considered that these BMP and BMP/FGF states may be only subtly distinct in their transcriptional programs; however, transcriptional profiling and marker analysis revealed these can be considered a quite distinct and stable cell state. This is most clear from the expression of the interferon damage response signature. The significance of the IRDS expression signature^22^, in quiescent NSCs remains unclear. However, it is interesting that recent studies have identified a role for interferon in the induction of quiescence in the ageing brain^44^. It is also notable that Lloren-Bobadilla et al. noted a similar induction of an interferon gamma-associated pathway during injury responses^23^. Our findings suggest that this may not be through canonical IFN ligand-driven responses, but rather is a consequence of combined BMP and FGF signaling. It will be interesting to explore the role and significance of this set of these interferon target genes, and we would speculate they may have some ancestral role in the activation of stem cells, stress responses or cell cycle re-entry checkpoint controls. It is possible these gene targets are activated alongside *Lrig1* via JAK-mediated signals. The NSC state we have generated in vitro may therefore represent a distinct subset of NSCs that operate during regeneration or repair.

Using the Fucci2a cells we also noted clear differences between the dormant and primed quiescent cells. Recent studies in *Drosophila melanogaster* have suggested that quiescent neural progenitors can arrest at G2^48^. However, we did not identify Venus positive cells within either BMP or BMP/FGF. The key consequence of FGF signaling in the presence of BMP is therefore to be in a primed G0 state, defined by lower P27 expression. Additional in vivo studies with the Fucci reporter mice will be needed to understand how Cdt1 protein degradation and DNA replication licensing machinery are regulated in the dormant and activated quiescent states.

Recent fate-mapping studies of the adult mouse SVZ seem to suggest that NSCs can transit back and forth between these proliferative and quiescent states^26^ and are consistent with our present findings. Our in vivo transplantation data suggests long term engraftment and re-entry into their original developmental program is possible, even after long term in vitro expansion. The p-qNSCs, therefore retain a capacity to re-engage with their previous niche and reveal their differentiation potential. This has practical importance, as NSC lines are highly amenable to genome editing and engineering. Thus, it now becomes feasible to engineering sophisticated modification in vitro and subsequent transplantation to create chimeric mice with transgenic or engineered NSCs. The adult SVZ NSCs may therefore be experimentally manipulated in similar ways to ES cells and HSCs (i.e. captured ex vivo, experimentally manipulated, and then transplanted to a host animal).

LRIG1 was originally identified as a regulator of stem cell balance in other niches (skin and intestine). We noted that Codega et al. reported in their supplementary datasets that *Lrig1* mRNAs are enriched in quiescent NSCs based on single expression profiling^25^ and very recent publication showed *Lrig1* expression throughout all the lateral wall^29^. Consistent with those observations, we have found that LRIG1 is expressed for a subset of NSCs in vivo and is highly expressed in primed quiescence NSCs in vitro. CRISPR-mediated ablation of *Lrig1* in NSCs resulted in hyperproliferation and mutant NSCs fail to enter in quiescence. In vivo, transplanted mutant cells in the SVZ show similar engraftment than their control WT but they display higher proliferation. Elimination of LRIG1 in HES5 population resulted in enhanced proliferation in the SVZ. We found increased numbers of GFAP/LRC + and KI67 + , suggesting that LRIG1 is required to maintain the quiescence state, avoiding awaking quiescence NSCs, most likely through EGFR signaling. On the other hand, forced overexpression of LRIG1 in NSCs, triggered cell cycle exit. All these data indicate there is an ongoing balance between LRIG1 levels and EGFR signaling that regulates quiescence NSCs transiting into an activated and proliferative state. The findings reported here raise many further questions. Do the distinct NSC states contribute equally to homeostatic neurogenesis? Do some cells serve as a reservoir for brain repair? Are there regional differences in quiescence control by LRIG1 within the heterogeneous NSCs? What is the significance of the interferon response signature in the activation in vivo? How long can this primed state persist in vivo?

Our findings are also likely to be relevant to brain cancer, glioblastoma (GBM). GBMs are driven by cells with NSC characteristics, including both quiescent and proliferative compartments. Quiescent GBM stem cells, evade cytotoxic and anti-mitotic therapies, are through to underlie the regrowth of the tumor. It is noteworthy that genome-wide association studies have been identified a risk allele associated with *LRIG1* (rs11706832)^49^. We would speculate that the quiescent cells in GBM are maintained in a primed state, with high Lrig1 expression and is consistent with their elevated CD9 expression^50^ and high IRDS expression that is associated with tumor resistance therapy^51^, and our findings of a lack of strong cytostatic responses induced by BMP^52^.

In summary, we conclude that BMP cooperates with FGF signaling to prepare cells for cell cycle re-entry – forcing them into a primed quiescent state. These primed NSCs are non-cycling, but upregulate critical markers and pathways in preparation for the exit from quiescence. We identify an important functional role for LRIG1 in NSCs, as it allows levels of EGFR to be highly expressed, but limits the signaling activity, thereby priming cells in a primed G0 for rapid cell cycle re-entry. LRIG1 is therefore a gatekeeper for the exit from quiescence. As LRIG1 is a well-known quiescence regulator of stem cells in intestinal and skin stem cells that constrains receptor-tyrosine kinase signaling pathways^53,54^, this primed quiescent state may be a state used by other tissue stem cells to balance proliferation and quiescence.

## Methods

### Cell culture

NSCs were isolated from the young adult 6-8 weeks old SVZ and maintained in vitro in serum-free basal medium supplemented with N2 and B27 (Life Technologies), Laminin-1 (Sigma, 1 μg/ml) and growth factors EGF and FGF2 (Peprotech, 10 ng/ml each)^55^. All the experiments were done in early passage after passage 3. For colony-forming assay NSCs were plated at low density (1000 cells per well-6 multiwell plate). We scored the total number of colonies per well. A minimal number of 6 grouped cells were considered a colony. Treatments with BMP-4 and FGF2/ BMP-4 were for 3 days. BMP-4 (10 ng/ml, Peprotech, AF-120-05ET-100), FGF2 (10 ng/ml, Peprotech, # 100-18b, EGF (10 ng/ml, Peprotech, #315-09), Gefitinib (LKT laboratories, G1721). Differentiation induction, NSCs were plated in no clonal density with 10 ng/ml, FGF for 2 div and then 2% FCS for 5 div.

### Animals

Mice used in this study were young adults (6-8-week old). Mice of the *Fucci2A* strains were generously provided by Richard Mort (IGMM) and Bl6 strains were maintained at the core facility of the University of Edinburgh in accordance with UK regulations. For NSCs transplants, animals were anaesthetized with 100% w/w Inhalation vapor, liquid isofluorane. Animals were fixed in a stereotactic apparatus stereotactic apparatus Stoelting (SKU S51725) and an automatic injector KD Scientific product, Model KDS-311-CE, catalog number 78-9311UU. NSCs concentrated (∼1 × 10^6^/ul) were injected in a volume of 0.2ul with a Hamilton syringe (0.1 μl min^−1^) for 2 min. Stereotactic coordinates used were 0 Anterior, 0.7 mm Lateral, 2.5 mm Deep. For in vivo detection of slow proliferating cells we used EdU (invitrogen) is dissolved in PBS at 5 mg/ml. We inject (IP) 1 mg/20 g mouse, 200 ul once daily for 3 days. After 3 weeks, mice were sacrificed and EdU detected by Click it (Thermo Fisher C10337). All animal procedures were performed under UK Government Home Office approved Procedure Project License (PC0395462) and following approval of the local Animal Welfare and Ethical Review Body (AWERB).

### Electroporation in vivo

Plasmids were injected into the ventricle of isoflurane-immobilized Rosa26-tdTomatofl/fl pups at postnatal day 2 using an Eppendorf femtojet, followed by electroporation (5 square pulses, 50 msec/pulse at 100 V, with 850 msec intervals). DNA was prepared to a final concentration of 1ug/ul with 0.1% fast green prepared in saline (0.9% NaCl)^47^. Plasmids used for electroporation in vivo were *Hes5*-SpCas9wt-T2A-iCRE-*Lrig1*guideRNA or *Hes5*-SpCas9wt-T2A-iCRE-controlRNA. Sequence of the *Lrig1* guide, AAGGCGACTCTCAGCGCGGC.

### Immunocytochemistry

Animals were deeply anaesthetized and transcardially perfused with 4% paraformaldehyde in 0.1 M PBS and brains processed for vibratome sectioning (Leica VT1,000S vibratome Samples were blocked in 10% normal goat serum and 0.2% Triton X-100 in PBS for 1 h and incubated for 48 h in blocking buffer with the appropriate primary antibodies: GFP (1: 500, 13970 Abcam), BIII tubulin (1:200 Covance, MMS435P), GFAP (1:100, G3893, Sigma-Aldrich), GFAP (1:500, Z0334 DAKO) KI67 (1:100 Thermo RM9106), OLIG2 (1:200, AB9610, Millipore), SOX2 (1:100, AB5603, Millipore), NESTIN (1:10, Rat 401, Developmental Studies Hybridoma Bank), ID1 (1;100, Biocheck, bch 1/37-2), LRIG1 (1:100, R&D, AF3688), S100B (1:100, Dako Z0311, 1:100), CD9 (1:100, 14-0091-82, eBioscience, 1:500), O4 (1:100, R&D MAB1326), p27 (1:200, sc-1641, Santa Cruz), PCNA (1:200, sc-56, Santa Cruz), RFP (1:500, Abcam 62341), FLAG M2 (1:1000, Sigma F1804).

Cells were fixed with 4% paraformaldehyde for 20 min, incubated in blocking buffer (10% normal goat serum and 0.2% Triton X-100 in 0.1 M phosphate buffer saline) for 30 min, and incubated overnight at 4 °C with the indicated primary antibodies. After several washes with PBS, immunoreactivity was detected appropriate Alexa Fluor-conjugated (Life Technologies) secondary antibody (1:500) diluted in blocking buffer. Cells were counterstained with 4′,6′, -diamidino-2-phenylindole (DAPI) or DRAQ5 and mounted with Fluorsave (Calbiochem).

Manufacturer’s instructions were followed for both EdU (Click it Thermo Fisher (C10337) and for protein synthesis (Click it Thermo Fisher OP-Puro (C10456)) assays. Images were taken and analyzed using Confocal (Leica SP8, 4 and 5 detectors) and Nikon TiE. For quantification of OP-Puro each cell was measured using Fiji and negative control with no fluorescence was used as negative control. For quantification of the immunofluorescent in vivo signals in Fucci in each cell were measured with Fiji as mean pixel (px) density as follows: above background, 5 px; mCherry high ≥ 50 px; mCherry low = 4–50 px; and mCherry negative ≤ 4 px. For CD9 levels quantification, we used same procedure above background, 7 px; CD9 high ≥ 60 px; CD9 low = 7–60 px; and CD9negative ≤ 6 px. Fiji version:2.0.0-rc-69/1.52i.

### Flow cytometry and sorting

For cell sorting using antibodies, we splited the cells and washed with PBS. Pellet of cells was incubated with the primary antibodies LRIG1 (1:200, R&D AF3688) and CD9 (1:500, eBioscience 14-0091) diluted in PBS with 4%FCS in the incubator at 37 °C for 1 h. After we washed with PBS to remove primary antibodies, secondary Alexa Fluor-conjugated (Life Technologies) antibodies diluted (1:500) in PBS-4%FCS were incubated for 10 min. When we sorted Fucci line, after the incubation of the antibodies we selected the different populations based on the levels in intensity of red mCherry (high and low). In both populations (high and low mCherry) we isolated more positive CD9 cells and plated for colony-forming assay and RNA extraction. We used BD LSR Fortessa (SORP) and BD Fusion Cell Sorter for analysis and sorting respectively.

### Western Immunoblotting

Immunoblotting was performed using standard protocols. Antibodies were diluted in 5% milk powder in PBS Triton 0.1%, and protein detection was carried out with HRP-coupled secondary antibodies and X-ray films. The following primary antibodies were used: LRIG1 (1:100, R&D, AF3688), EGFR (1:1000, D38B1, Cell Signaling, #4267), EGFR-p (1:1000, Tyr 1068, Cell Signaling, #3777), pSMAD1-5 (1:1000, Cell Signaling, #9516), SMAD1 (1:1000, Cell Signaling, #9743), ppERK1/2 (1:1000, Cell Signaling #9101), ERK1/2 (1:1000, Cell Signaling 4695), Phospho-AKT (Ser473) (1:1000, Cell Signaling #9271), AKT (1:2000, CST #9272), SOX2 (1:1000, Abcam #92494), GAPDH (1:50000; GenTex, GTX627408). β-ACTIN (1:5000, Sigma #A5316). No accutase was used for splitting the cells from the plate.

### Transfection and derivation of clonal lines

For the generation of eGFPp27-mCherryPCNA NSC line, guide RNA was manually designed to introduce the eGFP tag into the 5′ UTR of PCNA or mCherry tag into the 3′ UTR of CDKN2A (p27) based on the annotation of the 5′UTR, initial coding exon, final coding exon, and the 3′ UTR sequence. A ∼100 bp sequence around the stop codon was used as an input for guide RNA design using the DESKGEN cloud design tool. High-scoring guide RNAs were chosen based on minimal predicted off-target cleavage events and having a cut site within the 5′ UTR (PCNA) or 3′ UTR (CDKN2A/p27). For dsDNA block design, the eGFP/mCherry tag sequence was flanked by ∼200 bp homology arms and a PAM blocking mutation (NGG > NGC) was introduced to prevent re-cutting of donor DNA by Cas9. Ribonucleoprotein (RNP) was assembled and transfected^56^. Synthetic Alt-R crRNA and tracrRNA were manufactured by Integrated DNA Technologies. dsDNA block oligonucleotides were manufactured by Twist Bioscience and recombinant Cas9 protein was made in-house. Sequence of the guide RNA for p27, ACGTTTGACATCTTCCTCCT and for PCNA, GCGTGCCTCAAACATGGTGG. NSCs were transfected using PiggyBac-GFP-Luc BSD plasmid. For *Lrig1* mutant cells, design and construction of CRISPR sgRNAs are described in^33^. We disrupted Exon1 based in Suzuki et al. We used 4D Amaxa nucleofector and DN-100 program. For *Lrig1* deletion we used 2 RNA guides with the following sequences: AAGGCGACTCTCAGCGCGGC and TACTCACAGGCTGCGCGTCC. To overexpress *Lrig1* we used m*Lrig1*-CFLAG (Mouse LRIG1 expressed from CMV promoter; gift from Prof Kim Jensen, University of Copenhagen). See Supplementary Table 1 primer sequences.

### PCR-based Lrig1 genotyping cells

For genomic DNA isolation, each well of a confluent 24-well plate was lysed with 40 µl of lysis buffer (0.45% NP40, 0.45% Tween20, 1x NEB LongAmp PCR buffer) containing 0.2 mg ml−1 proteinase K (Sigma). After a 2 h digestion at 55 °C, samples were heated to 95 °C (10 min) and 1–2 µl of the lysate was used in a 10 µl PCR reaction. PCR mix consisted of 0.2 µl DMSO (100% v/v, Sigma), 0.3 µl dNTPs (10 mM, Thermo Fisher Scientific), 2.0 µl 5x LongAMP buffer (NEB), 0.4 µl LongAMP Taq DNA polymerase (NEB), and 12 pmol of each primer. Thermal cycling was performed using the following conditions: 1 cycle 94 °C for 3 min; 35 cycles (94 °C for 30 s, 60 °C for 30 s, 65 °C for 2 min); followed by a final extension at 65 °C for 10 min. Amplicon around 1200 bp (see Supplementary Table 1 primers sequences).

### Quantitative real-time RT-PCR

RNA was extracted using the RNeasy spin column kit (Qiagen), plus DNase treatment to eliminate gDNA. cDNA was generated with SuperScript III (Invitrogen), and quantitative RT-PCR was performed using Taqman Universal PCR Master Mix (Applied Biosystems). The following Taqman assays (Life Technologies) were used: *Egfr* (Mm00433023_m1, *Hes5* (Mm00439311_g1), *Tlx* (Mm00455855_m1), *mcm2* (Mm00484815_m1), *Sox2* (Mm03053810_s1), *Prom1* (Mm00726334_s1), *Bmi1* (Mm03053308_g1), *FoxG1* (Mm02059886_s1), *Gfap* (Mm99999915_g1), *p21* (Mm04205640_g1), *Nfix* (Mm00477791_m1), *Pdgfra* (Mm00440701_m1), *Ephrin2* (Mm01215897_m1), *Foxo3* (Mm00490673_m1), *Id1* (Mm00775963_g1), *Sox9* (Mm0083422_m1,) *Gli3* (Mm0049654_m1), *Gli1* (Mm00492345_m1), *Fabp7* (Mm00445225_m1), *Lrig1* (Mm00456116_m1), *S100* (Mm0048597), *Aqp4* (Mm00802131).

### RNA-seq processing and analysis

RNA extraction was performed using the Qiagen RNeasy Plus minispin column kit, eluting in 50 µL of RNase-free water, and using an additional DNase step. RNA concentration was determined using the Qubit RNA High-Sensitivity kit (Life Technologies). Sample concentration and quality was assessed using the Aligent Tapestation and Nanodrop, only samples with a RIN value of 9.6 or above were used. For RNA-seq we used RNA-Seq Quantification Library (Low input Library) BGISEQ-500RS. Fastq files were trimmed via Trimgalore (version 0.5.0) and aligned with Kallisto (version 0.44.0) to the mm10 transcriptome (Martin, 2011; Bray *et al*., 2016). Tximport (version 1.8.0) was used to summarize read counts before subsequent normalization and differential expression as per DESeq2 (version 1.27.32). All downstream analysis and visualization were completed following regularized logarithm transformation (rlog) via DESeq2. GO enrichment analysis was completed via the hypergeometric test using the R package clusterprofiler (version 3.15.4) on differentially expressed genes (> 1.5 log_2_ fold change, < 0.05 FDR) (Yu *et al*., 2012).

### RPPA analysis

For RPPA we prepared the samples Lysis Buffer: 1% Triton X-100, 50 mM HEPES, pH 7.4, 150 mM NaCl, 1.5 mM MgCl2, 1 mM EGTA, 100 mM NaF, 10 mM Na pyrophosphate, 1 mM Na3VO4, 10% glycerol, containing freshly added protease and phosphatase inhibitors from Roche Applied Science Cat. #: 05056489001 and 04906837001, respectively. 4x SDS Sample Buffer: 40% Glycerol, 8% SDS, 0.25 M Tris-HCL, pH 6.8. Before use, we added 2-mercaptoethanol at 1/10 of the volume. Clarified supernatants in biological triplicate were adjusted to 2 mg/mL concentration and printed onto nitrocellulose-coated slides (Grace Bio-Labs) in a dilution series (four serial 2-fold dilutions) in technical triplicate using an Aushon 2470 arrayer (Aushon Biosystems). Slides were blocked, probed with validated primary antibodies and detected with DyLight 800-conjugated secondary antibodies (New England BioLabs). Slides were read using an InnoScan 710-IR scanner (Innopsys) and quantified using Mapix (Innopsys). Relative fluorescence intensities were normalized to respective FastGreen-stained spots (total protein), and data were computationally analyzed.

### Statistic methods

Results are presented as mean ± SEM of a number (*n*) of independent experiments. Statistical significance was determined by two-tailed Student’s t-tests using GraphPad (version 9.0.0). Treatment experiments were analyzed by paired *t*-test. When comparisons were performed with relative values (normalized values and percentages), data were normalized by using an arc-sen transformation. Values of *P* < 0.05 were considered statistically significant. Box and whisker plots show the mean, and maximum and minimum values.

### Reporting summary

Further information on research design is available in the Nature Research Reporting Summary linked to this article.

## Supplementary information

Supplementary Information

Reporting Summary

## Data Availability

The datasets generated during and/or analyzed during the current study are available from the corresponding author on reasonable request and will be deposited in the GEO database (GSE168189). The remaining data are available within the Article, Supplementary Information or available from the authors upon request. Source data are provided with this paper.

## Source data

Source Data
