# Supplementary material for: LRIG1 is a gatekeeper to exit from quiescence in adult neural stem cells
Source: Nat Commun. 2021 May 10;12:2594. doi: 10.1038/s41467-021-22813-w (PMC8110534; doi:10.1038/s41467-021-22813-w)
Supplement: Supplementary file 1 — Supplementary Information [file 41467_2021_22813_MOESM1_ESM.pdf]

Supplementary Figures

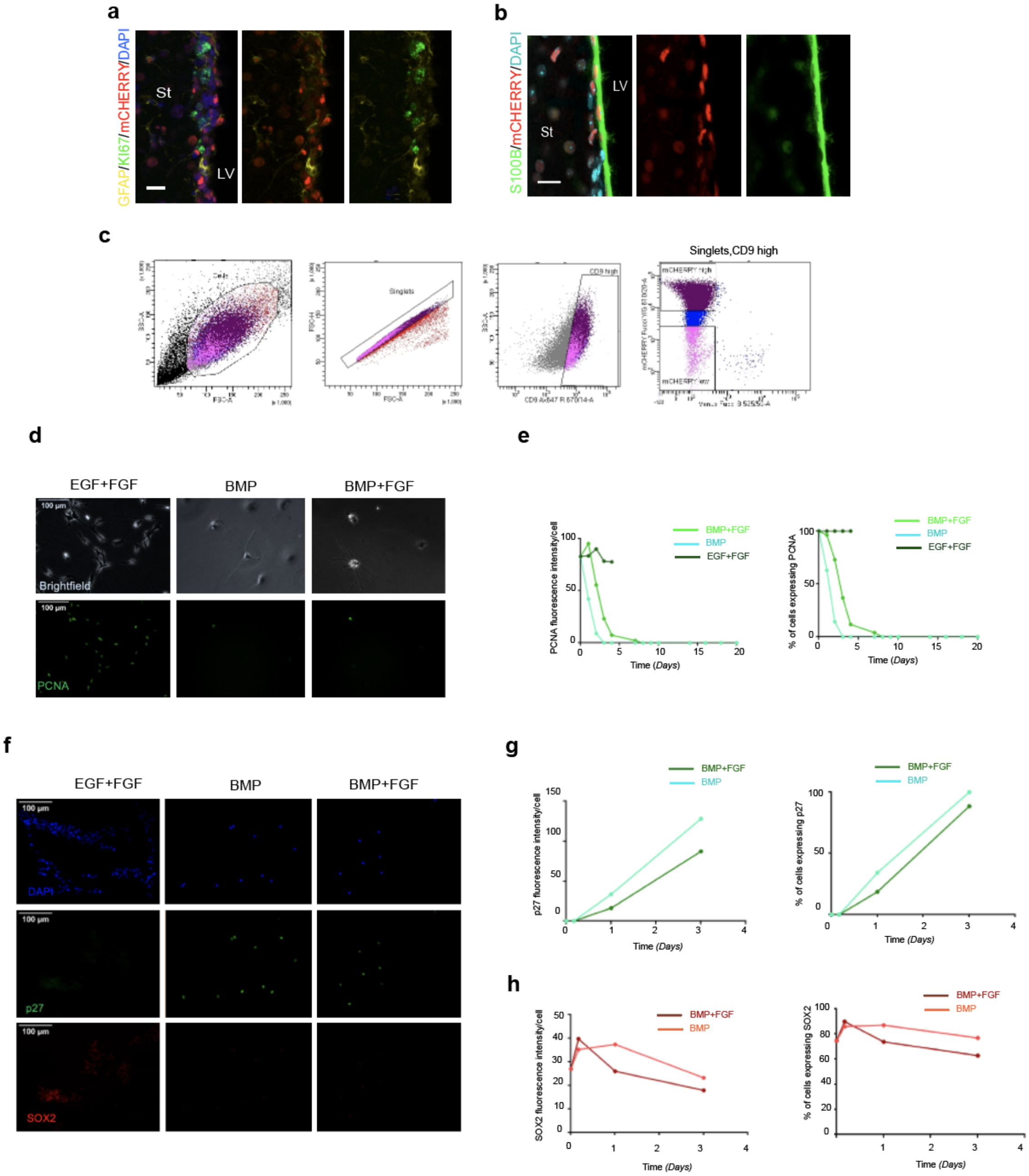

Figure S1 | Live cell imaging of a PCNA-GFP reporter NSC line. (a) Immunostaining of GFAP (yellow), KI67 (green) and mCHERRY (red) in SVZ of Fucci2a mice. (b) Immunostaining for S100B (green), mCHERRY (red) and nuclear counterstaining with DAPI (blue). (c) Gating strategy to sort high population of CD9 with different levels of mCHERRY reporter in NSCs. (d) Live cell imaging of PCNA of cells cultured with BMP and BMP/FGF for 4 days. (e) (left) Average PCNA fluorescence intensity per cell and percentage of cells expressing PCNA in cells cultured with BMP and BMP/FGF2 for different days (right) Quantification of the percentage of PCNA positive cells (n=3). (f) Immunocytochemistry assay reveals that p27 quiescence marker expression is notably increased with BMP and BMP/FGF2 nourishment while SOX2 stemness marker is very slightly decreased (n=3). (g) and (h) Variation in p27 and SOX2 expression and DAPI marker of cells cultured in proliferating medium, BMP and BMP/FGF2 for 3 days. Scale bar in (a) and (b) is 20 µm and in (d) and (f) is 100 µm. LV: Lateral Ventricle.

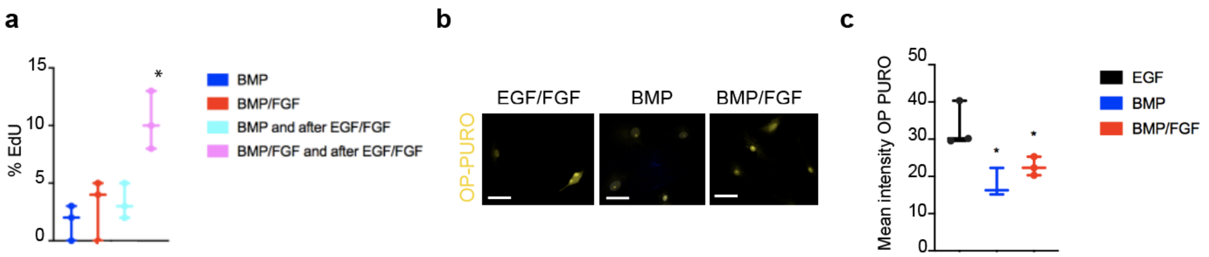

Figure S2 | Quiescent cells have reduced protein synthesis. (a) Percentage of EdU positive cells during treatments (left) NSCs in BMP and BMP/FGF don't incorporate EdU and afer to re-exposure to the mitogens (right). (n=3). (b) Images of the detection of protein synthesis using Click-iT® OPP Alexa Fluor® 647 in the different conditions in vitro. (c) Quantification of the mean intensity of OP-Puro. 500 cells were analyzed per condition. Scale bar in (b) is 20 um (n=3). Data are shown as mean ± SEM of the indicated number of the experiments (n) (* $p < 0.05$). Source data are provided as a Data Source File.

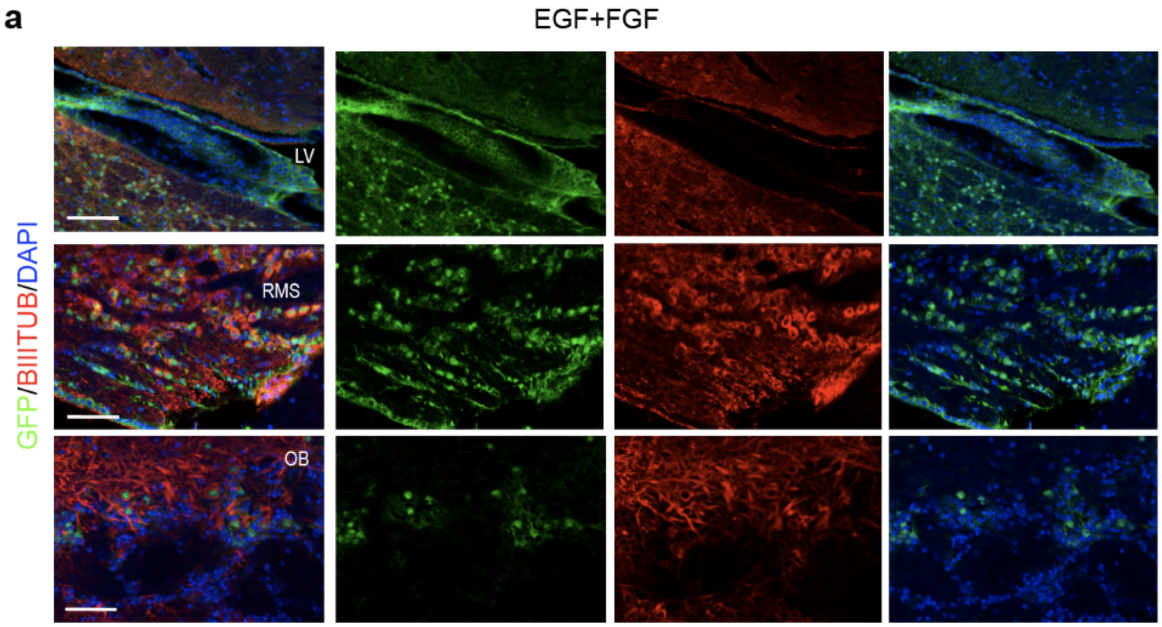

Figure S3 | Neural stem cells growing in vitro for several months are able to engraft in the adult SVZ and generate neurons in the olfactory bulb. One month after transplantation: (a) Panoramic immunostaining of NSCs engrafted in the SVZ (top panel), migrating through the RMS and arriving to the OB. GFP (green), BIII tubulin (red) and nuclear counterstaining with DAPI (blue). LV: lateral ventricle, RMS (rostral migratory stream) and OB (olfactory bulb). Scale bar in (A) is 100 um.

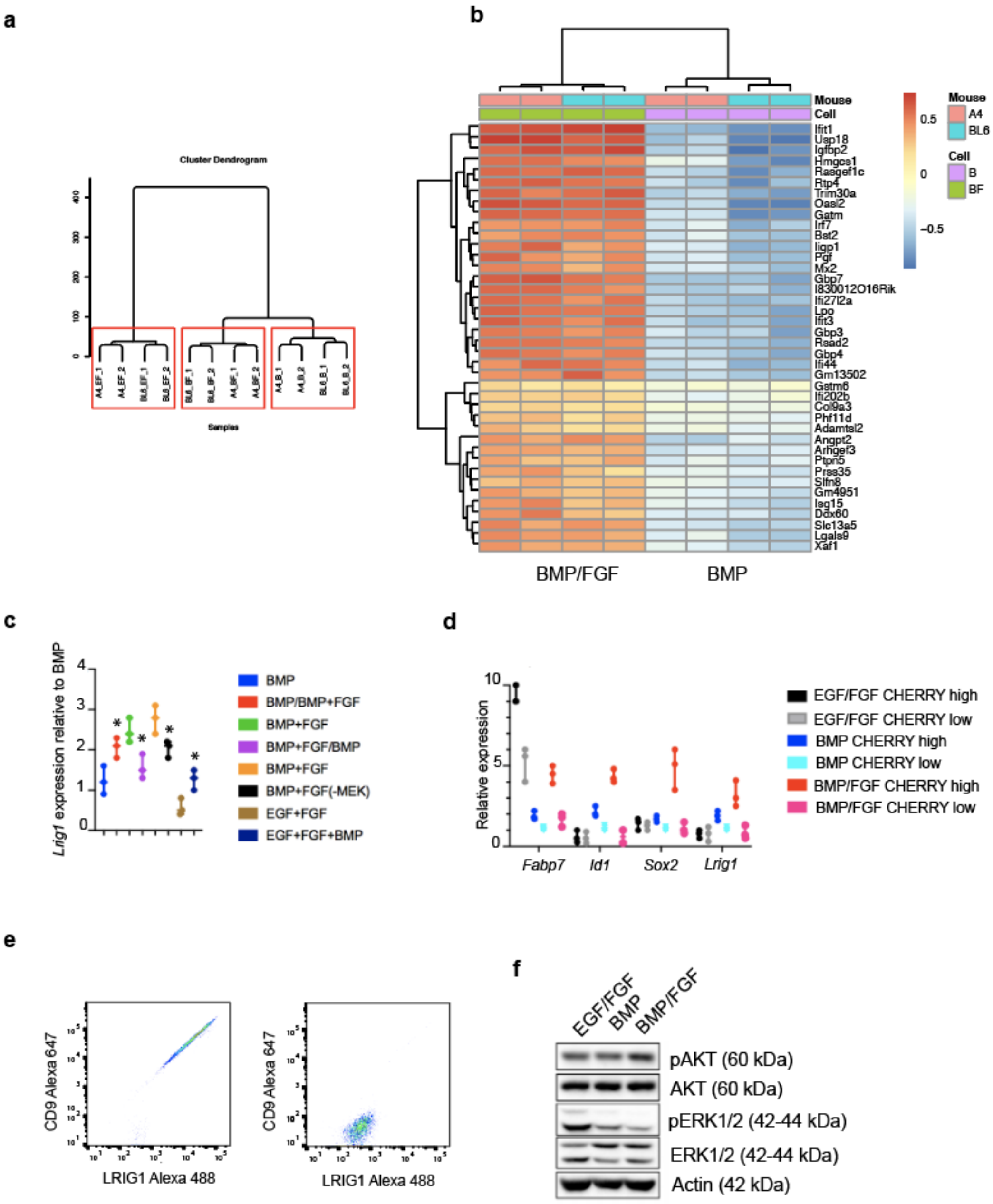

Figure S4 | Interferon type I is upregulated in quiescence population. (a) RNAseq analysis of the different states of NSCs. Cluster dendrogram showing the different NSCs lines (ANS4 and Bl6) in the different conditions (EGF+FGF2, BMP, BMP+FGF2). Number of biological replicas in RNA seq is 2. (b) Heat map of the most differentially expressed transcripts across RNA sequencing (RNA-seq) libraries in BMP and BMP+FGF2 using different NSC lines (ANS4 and Bl6). (c) *Lrig1* expression measured by qPCR in different conditions (n=3). Treatments were switched between BMP and BMP+FGF respectively after 3 days. MEK inhibitor was added to figure out *Lrig1* expression in quiescence condition. Control of EGF+FGF+BMP was added (d) Relative expression of *Fabp7, Id1, Sox2, Lrig1* of the sorted populations based in high levels of CD9 and different levels of mCHERRY Fucci reporter (n=3). (e) Cytometry analysis showing co-expression of LRIG1 (Alexa 488) and CD9 (Alexa 647) in cells treated with BMP/FGF. Right plot negative control of the same cells only with secondary antibodies. (f) WB for pAKT, AKT, pERK1/2, ERK and actin in the different conditions (n=3). Data are shown as mean ± SEM of the indicated number of the experiments (n) (*p < 0.05). Source data are provided as a Data Source File.

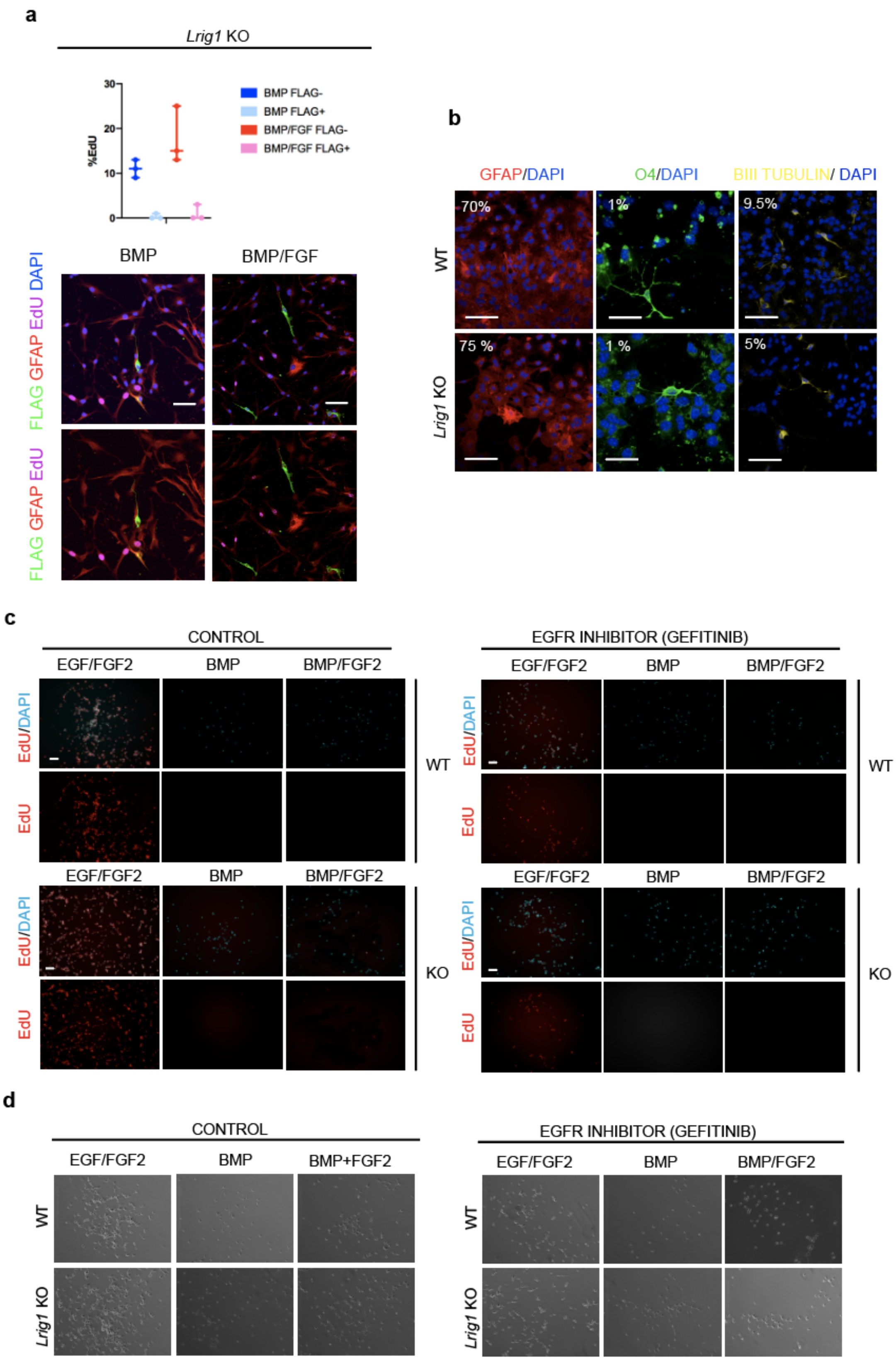
a
Lrig1 KO
30
20
10
0
%EdU
BMP FLAG-
BMP FLAG+
BMP/FGF FLAG-
BMP/FGF FLAG+
BMP
BMP/FGF
FLAG GFAP EdU DAPI
FLAG GFAP EdU
b
GFAP/DAPI
O4/DAPI
BIII TUBULIN/ DAPI
WT
Lrig1 KO
70%
1%
9.5%
75 %
1 %
5%
c
CONTROL
EGFR INHIBITOR (GEFITINIB)
EGF/FGF2
BMP
BMP/FGF2
EdU/DAPI
EdU
WT
KO
d
CONTROL
EGFR INHIBITOR (GEFITINIB)
EGF/FGF2
BMP
BMP+FGF2
BMP/FGF2
WT
Lrig1 KO

Figure S5 | Differentiation responses in *Lrig* null NSCs. (a) (Top) Quantification of EdU in *Lrig1* KO cells treated with BMP and BMP/FGF after transfection with m*Lrig1*-FLAG (n=3 independent experiments in each group). (Bottom) ICC for Flag (green), GFAP (red), EdU (magenta) and DAPI (blue) (n=3 per condition). (b) ICC for GFAP for astrocytes (red), O4 for oligodendrocytes (green) and BIII TUBULIN for neurons (yellow) in WT and *Lrig1* KO. Nuclear counterstaining with DAPI (blue) (n=3). Quantification of the percentage of cells. (c) EdU detection (red) and nuclear counterstaining with DAPI (blue) in the treated WT and *Lrig1* KO cells with and without EGFR inhibitor (n=3). (d) Phase contrast images of the cells WT and *Lrig1* KO in the different conditions with and without Gefitinib (EGFR inhibitor) (n=3). Scale bar in (a) and (b) is 50 um and in (c) is 100 um. Source data are provided as a Data Source File.

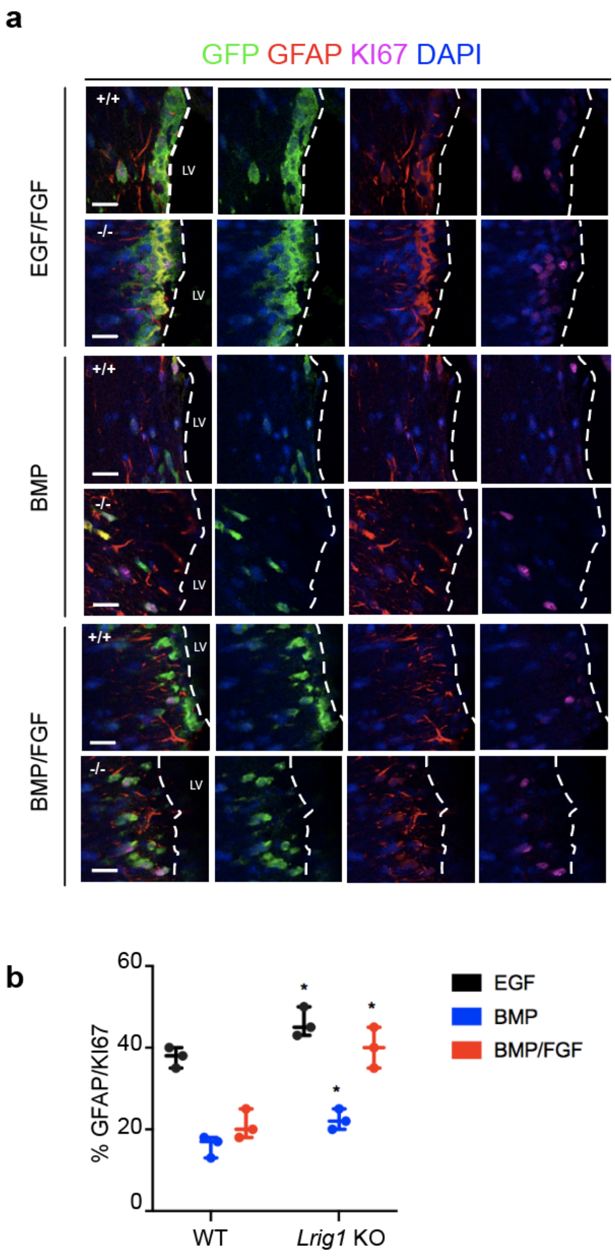

Figure S6 | *Lrig1* deficient cells display excessive proliferation in vivo. (a) Immunohistochemistry for GFP (green), GFAP (red), KI67 (magenta) and nuclear counterstaining with DAPI (blue). WT and KO cells were treated with EGF/FGF, BMP and BMP/FGF for 3 days before to be transplanted. LV: Lateral Ventricle. (b) Quantification of percentage of GFAP-KI67/GFP. Scale bar in (a) is 20 um. Data are shown as mean ± SEM of the indicated number of the experiments (n) (n=3) (*p < 0.05).

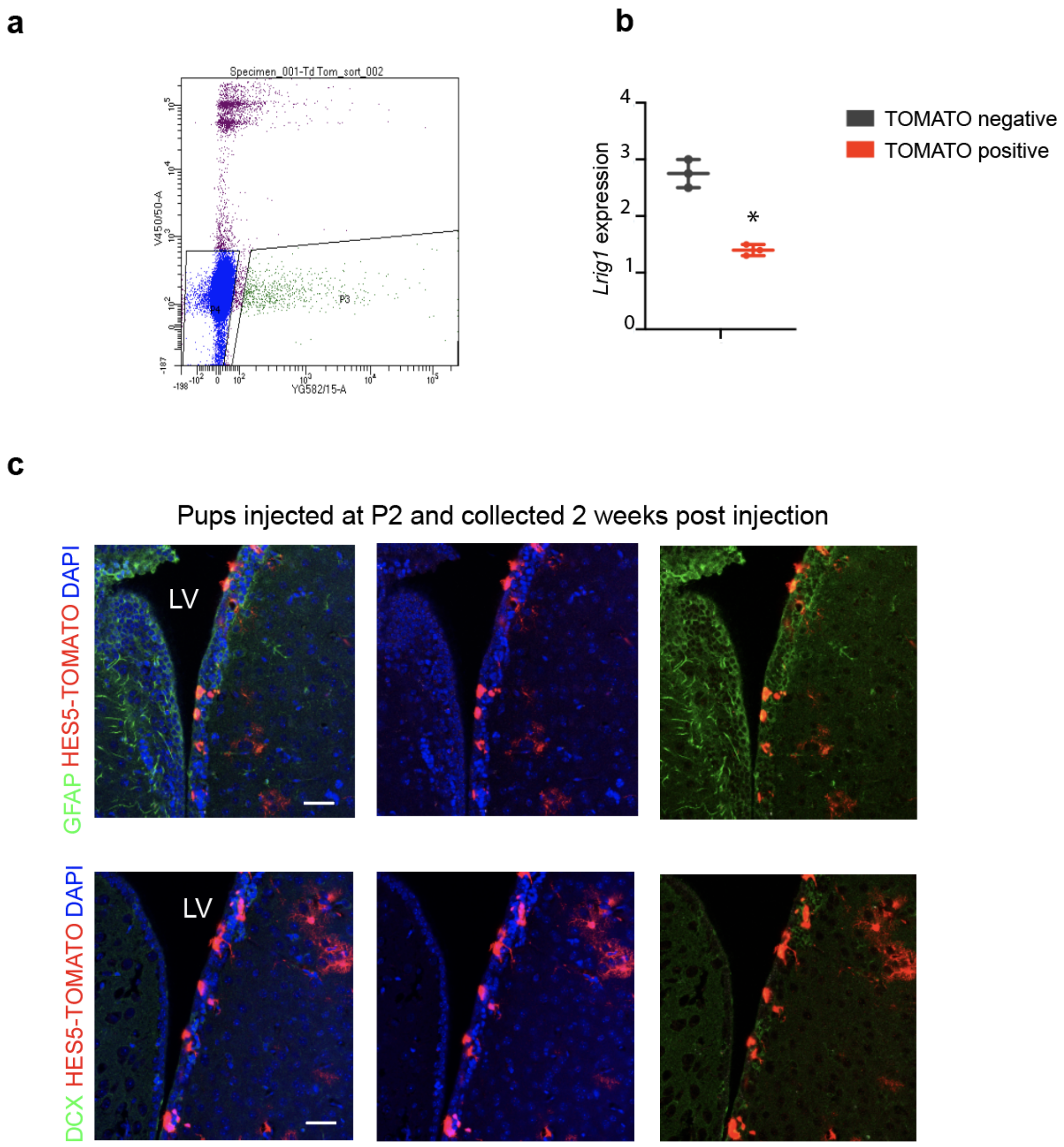

Figure S7 | HES5-TOMATO NSCs transfection in vitro and in vivo. (a) Validation of the guide for *Lrig1* deletion. Flow sorting analysis after transfection of NSC floxed tomato cells. (b) *Lrig1* expression by qPCR in the sorted population TOMATO- and TOMATO+ (n=3 per condition). (c) IHC of the SVZ of the Pups after 2 weeks post injection. HES5 TOMATO (red), GFAP (green), DCX (green) and nuclear counterstaining with DAPI (blue). LV: Lateral ventricle. Scale bar is c) is 30 um (*$p < 0.05$). Source data are provided as a Data Source File.

Supplementary Table 1. Primer sequences

	**Primer sequences**
Lrig1 Forward 1	5' CGAGCGCTCTTATGGGTTAG
*Lrig*1 Forward 2	5' TATGGGTTAGGACGCCAAAA
Lrig1 Reverse 1	5' CACACTCGCTGACTTCCAGA
Lrig1 Reverse 2	5' CACTCATGTCCTGCATCCTC
PCNA Forward	5' TGCTCAAACCACGGGTACGA
PCNA Reverse	5' CCCGCTCACCTGGTGAGGTT
p27 Forward	5' TTTTTCCGGAGAGAGGCGAGG
p27 Reverse	5'AGCTGCCCCTCTCCACCTCC
