# Supplementary material for: LRIG1 is a gatekeeper to exit from quiescence in adult neural stem cells
Source: Nat Commun. 2021 May 10;12:2594. doi: 10.1038/s41467-021-22813-w (PMC8110534; doi:10.1038/s41467-021-22813-w)
Supplement: Supplementary file 2 — Reporting Summary [file 41467_2021_22813_MOESM2_ESM.pdf]

nature research

Corresponding author(s): Prof Steven M. Pollard

Last updated by author(s): Feb 24, 2021

Reporting Summary

Nature Research wishes to improve the reproducibility of the work that we publish. This form provides structure for consistency and transparency in reporting. For further information on Nature Research policies, see our Editorial Policies and the Editorial Policy Checklist.

Statistics

For all statistical analyses, confirm that the following items are present in the figure legend, table legend, main text, or Methods section.

n/a	Confirmed	
☐	☒	The exact sample size (n) for each experimental group/condition, given as a discrete number and unit of measurement
☐	☒	A statement on whether measurements were taken from distinct samples or whether the same sample was measured repeatedly
☐	☒	The statistical test(s) used AND whether they are one- or two-sided<br>*Only common tests should be described solely by name; describe more complex techniques in the Methods section.*
☒	☐	A description of all covariates tested
☐	☒	A description of any assumptions or corrections, such as tests of normality and adjustment for multiple comparisons
☐	☒	A full description of the statistical parameters including central tendency (e.g. means) or other basic estimates (e.g. regression coefficient) AND variation (e.g. standard deviation) or associated estimates of uncertainty (e.g. confidence intervals)
☒	☐	For null hypothesis testing, the test statistic (e.g. F, t, r) with confidence intervals, effect sizes, degrees of freedom and P value noted<br>*Give P values as exact values whenever suitable.*
☒	☐	For Bayesian analysis, information on the choice of priors and Markov chain Monte Carlo settings
☒	☐	For hierarchical and complex designs, identification of the appropriate level for tests and full reporting of outcomes
☒	☐	Estimates of effect sizes (e.g. Cohen's d, Pearson's r), indicating how they were calculated

Our web collection on statistics for biologists contains articles on many of the points above.

Software and code

Policy information about availability of computer code

Data collection	*Provide a description of all commercial, open source and custom code used to collect the data in this study, specifying the version used OR state that no software was used.*
Data analysis	Fiji (2.0.0-rc-69/1.52i.) and GrapNhoPtapdpl(ivcaebrlesion 9.0.0).

For manuscripts utilizing custom algorithms or software that are central to the research but not yet described in published literature, software must be made available to editors and reviewers. We strongly encourage code deposition in a community repository (e.g. GitHub). See the Nature Research guidelines for submitting code & software for further information.

Data

Policy information about availability of data

All manuscripts must include a data availability statement. This statement should provide the following information, where applicable:

- Accession codes, unique identifiers, or web links for publicly available datasets
- A list of figures that have associated raw data
- A description of any restrictions on data availability

The datasets generated during and/or analysed during the current study are available from the corresponding author on reasonable request.

Field-specific reporting

Life sciences study design

All studies must disclose on these points even when the disclosure is negative.

Sample size	All experiments with mice and cell line were performed at least in triplicate.
Data exclusions	We excluded some transplanted mice where there were technical with transplantation and no evidence of cells engrafted.
Replication	Three independent experiments were performed.
Randomization	na
Blinding	na

Reporting for specific materials, systems and methods

We require information from authors about some types of materials, experimental systems and methods used in many studies. Here, indicate whether each material, system or method listed is relevant to your study. If you are not sure if a list item applies to your research, read the appropriate section before selecting a response.

Materials & experimental systems

n/a	Involved in the study	
☐	☒	Antibodies
☐	☒	Eukaryotic cell lines
☒	☐	Palaeontology and archaeology
☐	☒	Animals and other organisms
☒	☐	Human research participants
☒	☐	Clinical data
☒	☐	Dual use research of concern

Methods

n/a	Involved in the study	
☒	☐	ChIP-seq
☐	☒	Flow cytometry
☒	☐	MRI-based neuroimaging

Antibodies

Antibodies used	GFP (1: 500, 13970 Abcam) , BIII tubulin (1:200 Covance, MMS435P), GFAP (1:100, G3893, Sigma-Aldrich), GFAP (1:500, Z0334 DAKO) KI67 (1:100 Thermo RM9106), OLIG2 (1:200, AB9610, Millipore), SOX2 (1:100, AB5603, Millipore),<br>NESTIN (1:10, Rat 401, Developmental Studies Hybridoma Bank), ID1 (1;100, Biocheck, bch 1/37-2), LRIG1 (1:100, R&D, AF3688), S100B (1:100, Dako Z0311, 1:100), CD9 (1:100, 14-0091-82, eBioscience, 1:500),<br>O4 (1:100, R&D MAB1326), p27 (1:200, sc-1641, Santa Cruz) , PCNA (1:200, sc-56, Santa Cruz), RFP (1:500, Abcam 62341), FLAG M2 (1:1000, Sigma F1804).
Validation	Validation was based on manufacturers datasheets, or was internally controlled in our experiments.

Eukaryotic cell lines

Policy information about cell lines

Cell line source(s)	Primary cell lines derived in this study were from adult SVZ tissue of young mice.
Authentication	na
Mycoplasma contamination	na
Commonly misidentified lines<br>(See ICLAC register)	na

Animals and other organisms

Policy information about studies involving animals; ARRIVE guidelines recommended for reporting animal research

Laboratory animals	Postnatal day 2 mice and adult mice.
Wild animals	*Provide details on animals observed in or captured in the field; report species, sex and age where possible. Describe how animals were caught and transported and what happened to captive animals after the study (if killed, explain why and describe method; if released, say where and when) OR state that the study did not involve wild animals.*

Field-collected samples	*For laboratory work with field-collected samples, describe all relevant parameters such as housing, maintenance, temperature, photoperiod and end-of-experiment protocol OR state that the study did not involve samples collected from the field.*
Ethics oversight	*Identify the organization(s) that approved or provided guidance on the study protocol, OR state that no ethical approval or guidance was required and explain why not.*

Note that full information on the approval of the study protocol must also be provided in the manuscript.

Flow Cytometry

Plots

Confirm that:

- [x] The axis labels state the marker and fluorochrome used (e.g. CD4-FITC).
- [x] The axis scales are clearly visible. Include numbers along axes only for bottom left plot of group (a 'group' is an analysis of identical markers).
- [x] All plots are contour plots with outliers or pseudocolor plots.
- [x] A numerical value for number of cells or percentage (with statistics) is provided.

Methodology

Sample preparation	We split the cells and washed with PBS. Pellet of cells was incubated with the primary antibodies diluted in PBS with 4%FCS in the incubator at 37C for<br>1 hour. After we washed with PBS to remove primary antibodies, secondary Alexa Fluor-conjugated (Life Technologies) antibodies diluted (1:500) in<br>PBS-4%FCS were incubated for 10 min.
Instrument	BD LSR Fortessa (SORP) and BD Fusion Cell Sorter for analysis and sorting respectively
Software	FlowJo software
Cell population abundance	na
Gating strategy	For isolation of Lrig1 ablated cells, we gated on parental non CRISPR treated control lines, shown in the main Figure.

- [x] Tick this box to confirm that a figure exemplifying the gating strategy is provided in the Supplementary Information.
